# Dissolution Study of Biodegradable Magnesium Silicide Thin Films for Transient Electronic Applications

**DOI:** 10.1002/advs.202518093

**Published:** 2025-11-25

**Authors:** Ji‐Woo Gu, Jun‐Seok Shim, Minjung Chae, Yoonseong Jung, Su‐Min Kim, Young‐In Ryu, Jae‐Hwan Lee, Sung‐Woo Kim, Kyung‐Sub Kim, Tae‐Woo Lee, Edyta Wyszkowska, Jungho Shin, Hyejin Jang, Ju‐Young Kim, Myoung‐Ryul Ok, Jong‐hyoung Kim, Jae‐Young Bae, Seung‐Kyun Kang

**Affiliations:** ^1^ Department of Materials Science and Engineering Seoul National University Seoul 08826 Republic of Korea; ^2^ Research Institute of Advanced Materials (RIAM) Seoul National University Seoul 08826 Republic of Korea; ^3^ Nano Systems Institute SOFT Foundry Seoul National University Seoul 08826 Republic of Korea; ^4^ Biomaterials Research Center Biomedical Research Division Korea Institute of Science and Technology (KIST) Seoul 02792 Republic of Korea; ^5^ Division of Bio‐Medical Science and Technology KIST School Korea University of Science and Technology (KIST) Seoul 02792 Republic of Korea; ^6^ Department of Materials Science and Engineering Ulsan National Institute of Science and Technology (UNIST) Ulsan 44919 Republic of Korea; ^7^ Graduate School of Semiconductor Materials and Devices Engineering Ulsan National Institute of Science and Technology (UNIST) Ulsan 44919 Republic of Korea; ^8^ National Centre for Nuclear Research NOMATEN CoE MAB+ ul. Andrzeja Sołtana 7 Otwock‐Swierk 05‐400 Poland; ^9^ Department of Advanced Materials and Biochemical Engineering Gangneung‐Wonju National University Gangwon‐do 25457 Republic of Korea; ^10^ Research Institute for Dental Engineering Gangneung‐Wonju National University Gangwon‐do 25457 Republic of Korea; ^11^ Department of Materials Science and Engineering Pukyong National University Busan 48513 Republic of Korea

**Keywords:** biodegradable photosensor, biodegradable semiconductor, biodegradable thermoelectric generator, magnesium silicide, transient electronics

## Abstract

Transient electronic systems offer compelling solutions for sustainable technologies, enabling environmentally benign disposal in ecological settings and eliminating surgical retrieval in biomedical implants. At the core of such systems, biodegradable semiconductors serve as key materials not only for logic operations but also for realizing diverse sensing modalities. Here, a comprehensive study of magnesium silicide (Mg_2_Si) thin films as a narrow‐bandgap, biodegradable semiconductor platform for transient electronics is reported. Polycrystalline Mg_2_Si thin films are formed via RF magnetron sputtering and thermal annealing, followed by systematic investigation of their dissolution behavior under various pH and ionic conditions. Physiological relevance is confirmed by phosphate‐buffered saline testing, while environmental biodegradability is validated under composting conditions. In vitro cytotoxicity assays confirmed the biocompatibility of the material and its degradation byproducts. Mg_2_Si thin films exhibit an indirect bandgap of ≈0.84 eV, intrinsic carrier concentration (>10^18^ cm^−3^), and thermal conductivity (<1.8 W m^−1^ K^−1^), along with broadband optical absorbance. Device‐level integration into thermoelectric harvesters yielded Seebeck coefficients of ≈130 µV K^−1^ and output power exceeding ≈0.338 µW cm^−2^ K^−2^. Photosensors demonstrated photoresponse up to 1300 nm, confirming near‐infrared sensitivity. These results establish Mg_2_Si as a viable semiconductor for transient electronics, expanding the material spectrum beyond conventional wide‐bandgap semiconductors.

## Graphical abstract

Magnesium silicide (Mg_2_Si) is introduced as a narrow‐bandgap, biodegradable semiconductor for transient electronics. RF‐sputtered and annealed Mg_2_Si thin films show high intrinsic electrical conductivity and low thermal conductivity. The polycrystalline material undergoes hydrolysis in aquatic and composting environments with minimal cytotoxicity. Functional prototypes are demonstrated including a biodegradable thermoelectric generator and a photosensor with Mg_2_Si thin films.

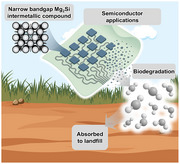

## 1 Introduction

Transient electronic systems,^[^ ^1^, ^2^, ^3^, ^4^, ^5^, ^6^, ^7^, ^8^, ^9^, ^10^, ^11^, ^12^ ^]^ capable of operating in physiological or natural environments and undergoing complete disintegration after use, have emerged as promising platforms for environmentally sustainable zero‐waste devices^[^ ^13^, ^14^, ^15^ ^]^ and minimally invasive implantable medical applications.^[^ ^2^, ^3^, ^16^ ^]^ The elucidation of hydrolysis behavior in electronic‐grade single‐crystal Si nanomembranes,^[^ ^17^, ^18^, ^19^, ^20^, ^21^ ^]^ together with the integration of biodegradable metals (e.g., Mg, Zn, W, Mo)^[^ ^12^, ^22^, ^23^, ^24^ ^]^ and insulating materials (e.g., SiO_2_, MgO),^[^ ^1^, ^16^, ^19^, ^25^, ^26^, ^27^, ^28^ ^]^ has played a key role in transforming conventional silicon‐based devices into fully transient systems. Leveraging its intrinsic semiconducting properties, degradable Si nanomembrane has been integrated into active devices such as diodes,^[^ ^1^, ^3^, ^8^, ^10^, ^11^, ^12^, ^14^, ^16^, ^19^, ^21^ ^]^ field‐effect transistors (FETs),^[^ ^9^, ^13^, ^19^, ^29^, ^30^ ^]^ CMOS,^[^ ^8^, ^9^, ^19^ ^]^ and logic circuits (e.g., NAND, NOR),^[^ ^8^, ^30^ ^]^ as well as temperature^[^ ^1^, ^2^, ^3^, ^31^ ^]^ and pH sensors,^[^ ^1^, ^3^, ^19^ ^]^ and multiplexed electrode arrays for biosignal acquisition.^[^ ^3^ ^]^ Furthermore, its photoconductive and photovoltaic characteristics have supported the realization of transient optoelectronic systems, such as optical sensors^[^ ^32^ ^]^ and solar cells.^[^ ^21^ ^]^ These devices have been further adapted for specific biomedical applications, including implantable intracranial pressure monitors based on piezoresistive Si nanomembrane gauges,^[^ ^2^ ^]^ wireless peripheral nerve stimulators using diode‐based rectifiers,^[^ ^10^, ^12^ ^]^ and optotransistor‐integrated pacemakers for cardiac electrotherapy.^[^ ^16^ ^]^

A detailed understanding of dissolution kinetics has laid a critical foundation for expanding the material palette of biodegradable semiconductors. In particular, silicon nanomembranes have served as a model system for characterizing environmentally triggered hydrolysis behavior under physiologically relevant conditions. The degradation rate of Si has been shown to depend strongly on environmental factors, including pH^[^ ^33^ ^]^ and ionic species.^[^ ^19^, ^20^, ^21^ ^]^ For instance, basic and chloride‐rich environments accelerate hydrolysis, with dissolution rates increasing from ≈2 nm day^−1^ in deionized water (pH 7)^[^ ^18^ ^]^ to ≈10 nm day^−1^ in buffer solution (pH 10)^[^ ^19^ ^]^ and ≈60 nm day^−1^ in 1 m NaCl solution^[^ ^20^ ^]^ at 37 °C. Phosphate‐buffered saline (PBS),^[^ ^20^ ^]^ fetal bovine serum,^[^ ^20^ ^]^ and seawater^[^ ^18^, ^33^ ^]^ have also been employed to mimic biological and environmental exposure scenarios, providing tunable degradation profiles through ion‐mediated surface chemistry. In parallel, intrinsic material parameters such as crystallinity and doping have been systematically investigated. Amorphous Si nanomembranes exhibit faster dissolution (≈4.1 nm day^−1^) than their polycrystalline and single‐crystalline counterparts (≈2.8  and ≈2.9 nm day^−1^ respectively) in PBS (pH 7.4) at 37 °C.^[^ ^21^ ^]^ The low packing density of a‐Si leads to an enhancement of water molecule penetration.^[^ ^21^ ^]^ Moreover, doping has been shown to modulate dissolution behavior: both boron and phosphorus dopants can reduce hydrolytic degradation rates by altering the electronic structure and chemical reactivity of the silicon surface.^[^ ^18^ ^]^

Building on the foundational understanding of silicon dissolution, research has expanded to encompass a broader set of inorganic semiconductors with distinct electronic and degradation properties. Germanium (Ge, bandgap (E_g_) ≈ 0.8 eV) dissolves at a rate of ≈3.1 nm h^−1^ in PBS at 37 °C, offering broadband absorption and high carrier mobility that make it suitable for infrared‐responsive and low‐power optoelectronic applications.^[^ ^21^ ^]^ Additionally, silicon germanium alloys (e.g., Si:Ge = 8:2, E_g_ ≈ 1.0 eV) exhibit hydrolytic dissolution at a moderate rate (≈0.1 nm day^−1^ in PBS at 37 °C), providing a means to tune degradation behavior while maintaining silicon‐compatible electronic characteristics.^[^ ^21^ ^]^ Amorphous indium gallium zinc oxide (a‐IGZO, E_g_ ≈ 3.1 eV), a widely studied compound semiconductor, displays composition‐dependent dissolution kinetics (e.g., 10–20 nm day^−1^ in DI water at 60 °C), along with favorable properties such as high optical transparency and moderate field‐effect mobility (≈10 cm^2^ V^−1^ s^−1^), enabling the fabrication of transparent, low‐power transient thin‐film transistors.^[^ ^34^ ^]^ Zinc oxide (ZnO, E_g_ ≈ 3.3 eV) degrades rapidly under mildly acidic conditions (>3 µm day^−1^ at pH 4–5) and has been employed in UV photosensors owing to its strong UV absorption, and in energy‐harvesting devices leveraging its piezovoltaic characteristics.^[^ ^35^, ^36^ ^]^ Further extending to low‐dimensional materials, chemical vapor deposited molybdenum disulfide (MoS_2_, E_g_ ≈ 1.81 eV) has demonstrated hydrothermal degradation at grain boundaries (e.g., up to 180 nm of morphological change over 8 days in PBS at 75 °C), highlighting its potential for use in 2D, near‐infrared (NIR) transient electronics.^[^ ^37^ ^]^

Biodegradable thermoelectric (TE) energy harvesting systems represent a critical advancement beyond conventional transient energy devices (batteries, photovoltaics/piezoelectric devices, or supercapacitors) by enabling continuous and maintenance‐free power generation from ubiquitous thermal gradients. Building on this advantage, TE‐based powering devices without reliance on finite electrochemical reactants, external stimuli or spatiotemporal operation imparts their potential as a sustainable foundation for next‐generation self‐sustainable transient electronics. Magnesium silicide (Mg_2_Si) represents a promising expansion to the current library of biodegradable semiconductors, offering complementary properties that expand the functional design space of transient electronic materials. As a binary compound composed of environmentally benign elements, Mg_2_Si exhibits a narrow indirect bandgap of ≈0.6–0.8 eV and has been widely investigated as an n‐type thermoelectric material owing to its high electrical conductivity (>10^3^ S cm^−1^), moderate Seebeck coefficient (≈200 µV K^−1^), and intrinsically low thermal conductivity (<10 W m^−1^ K^−1^).^[^ ^38^, ^39^, ^40^, ^41^, ^42^ ^]^ Beyond its thermoelectric function, Mg_2_Si nanoparticles have been explored as biocompatible deoxygenation agents in cancer therapy, highlighting their potential for bio‐integrated applications.^[^ ^38^ ^]^ This combination of favorable electronic characteristics and safe, predictable biodegradability positions Mg_2_Si as a key material platform for eco‐friendly and waste‐free transient electronics, particularly in TE energy harvesting and broadband optical sensing systems that demand both electronic functionality and environmental compatibility with sustainable operation.

Here, we report a comprehensive dissolution study of Mg_2_Si thin films, establishing its potential as a low‐bandgap, biodegradable semiconductor platform for transient electronics. The polycrystalline Mg_2_Si thin films, a binary intermetallic phase, were formed via RF magnetron sputtering followed by thermal annealing. The hydrolysis behavior of Mg_2_Si was systematically investigated under various ionic and pH conditions, revealing that degradation kinetics are strongly accelerated in acidic environments and in the presence of chloride ion, while decelerated in phosphate‐rich conditions. To assess its suitability for bioresorbable applications, further dissolution studies were conducted in phosphate‐buffered saline (PBS), a physiologically relevant medium. In vitro cytocompatibility was evaluated by measuring the viability of L929 fibroblast cells cultured on Mg_2_Si thin films, confirming high biocompatibility of both the material and its degradation byproducts. Environmental biodegradability was also assessed by monitoring the degradation of Mg_2_Si‐based optoelectronic devices in a composting environment. The electronic and thermoelectric properties were investigated by using the Hall effect and Seebeck coefficient measurement, respectively. Furthermore, optoelectronic properties were characterized over a broad spectral range in UV–vis absorbance and photoelectron spectroscopy. Together, these results establish Mg_2_Si as a versatile and biodegradable semiconductor platform that combines low‐bandgap optoelectronic functionality with environmental and biological compatibility, thereby broadening the material spectrum for transient electronics.

## 2 Results and Discussion

**Figure 1:**
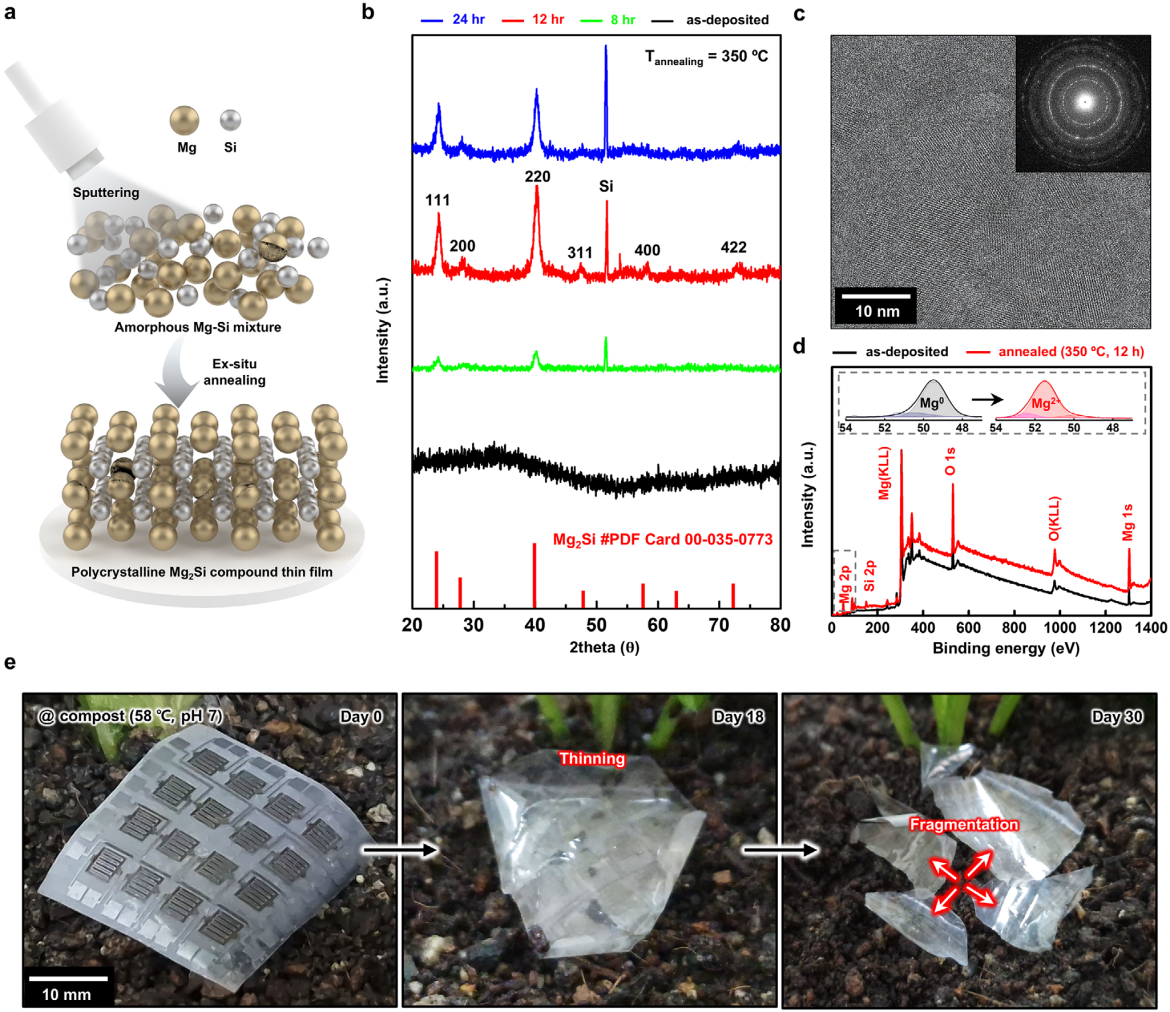
Characterization of the Mg_2_Si thin films and demonstration of device‐scale degradation. a) Schematic diagram of the fabrication process of the Mg_2_Si thin films prepared by sputtering and thermal annealing. b) X‐ray diffraction patterns of the Mg_2_Si thin films under annealing time variations at 350 °C (as‐deposited, black; 8 h, green; 12 h, red; 24 h, blue). c) High‐resolution transimission electron microscopy image of polycrystalline phase of the Mg_2_Si thin films annealed at 350 °C for 12 h (Inset: selected‐area electron diffraction patterns). d) Photoelectron spectrum of the (black) as‐deposited Mg─Si and (red) annealed Mg_2_Si thin films (350 °C, 12 h) (Inset: elemental deconvoluted spectrums of Mg 2p peak measured before and after annealing. e) Photographs of device degradation of the Mg_2_Si thin film‐based photosensor arrays undergoing degradation in a composting environment (58 °C, pH 7) at day 0 (left), day 18 (middle), and day 30 (right).

Biodegradable Mg_2_Si thin films were prepared by magnetron sputtering an Mg_2_Si target and thermal annealing (**Figure** **1**a). An amorphous mixture of Mg and Si atoms was initially formed on a Si(100) wafer. Annealing was performed to induce crystallization and obtain Mg_2_Si thin films. X‐ray diffraction (XRD) patterns, transmission electron microscopy (TEM) observation, and X‐ray photoelectron spectroscopy (XPS) analysis confirmed the formation of a polycrystalline phase in the Mg_2_Si thin films after annealing. XRD patterns (Figure 1b) reveal that the initially amorphous Mg─Si thin films undergo progressive crystallization as annealing time increased from 0 to 24 h at 350 °C. These peaks became more intense after 12 h of annealing, corresponding to the antifluorite crystal structure of Mg_2_Si, as previously reported in the literature.^[^ ^43^, ^44^ ^]^ The crystallite size of the Mg_2_Si thin films increased rapidly from ≈28 to 36 nm between 4 and 12 h of annealing, after which further growth was minimal, indicating saturation of crystallite coarsening under the given thermal conditions (Figure S1 and Note S1, Supporting Information). High‐resolution transmission electron microscopy (HRTEM) image and selected area electron diffraction (SAED) pattern (Figure 1c) confirmed the formation of a polycrystalline phase of Mg_2_Si thin films after 12 h annealing. The interplanar spacing of 0.44 nm observed in the annealed sample (350 °C, 12 h) corresponded to the (110) planes of the antifluorite structure of Mg_2_Si (Figure S2, Supporting Information). Thermal annealing above the optimal point at 350 °C induces a phase transition in the Mg_2_Si thin films, as evidenced by the diffrential scanning calorimetry (DSC) and XRD analyses (Figure S3, Supporting Information). This phase transformation marks the onset of material degradation, indicating thermal instability under elevated annealing conditions.

XPS analysis in Figure 1d provides further evidence of Mg─Si bond formation. The deconvoluted Mg 2p spectrum shows a dominant peak at 49.8 eV in the as‐deposited state, corresponding to neutral Mg^0^ atoms. After annealing at 350 °C for 12 h, a new component was observed at 51.5 eV, assigned to Mg^2+^, indicating the formation of polar covalent Mg─Si bonds.^[^ ^45^ ^]^ In the as‐deposited film, Mg and Si exist as separate atoms, forming an amorphous network; upon annealing, they react to form a chemically bonded compound phase. Raman spectroscopy further supports Mg─Si bond formation in polycrystalline Mg_2_Si thin films by identifying vibrational modes. The as‐deposited films show broad, low‐intensity spectrum, while the annealed films exhibit sharp peaks corresponding to vibrational modes of Mg─Si bonds (Figure S4, Supporting Information). The peak at 255 cm^−1^ arose from transverse optical bending between Mg and Si atoms, and the 338 cm^−1^ peak corresponded to a longitudinal optical stretching mode, consistent with crystalline Mg_2_Si.^[^ ^46^ ^]^ Changes in crystallinity were also visualized in nanoindentation‐based hardness mapping, showing mechanical contrast that reflects the underlying structural evolution. As presented in Figure S5 and Note S2 (Supporting Information), the average hardness of the as‐deposited Mg─Si film was 5411.16 ± 626.36 MPa, whereas annealing increased the value to 7911.83 ± 293.68 MPa. The pronounced hardening, accompanied by the reduced deviation, suggests that the as‐deposited Mg─Si film possessed a relatively loose and heterogeneous structure, and that crystallization into a well‐ordered Mg_2_Si phase upon annealing enhanced atomic packing and reduced defects, thereby leading to the observed increase in hardness and improvement in uniformity.

**Figure 2:**
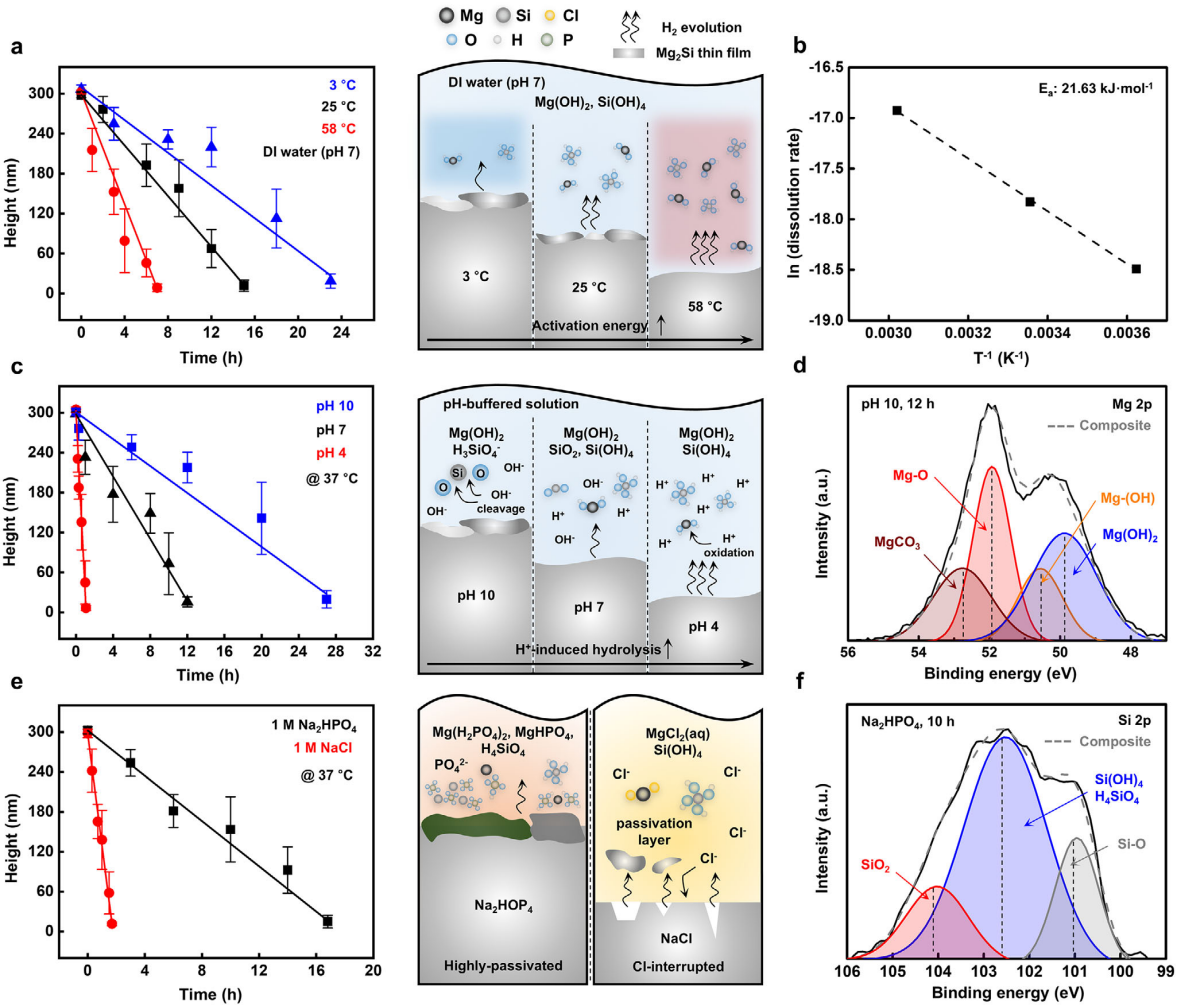
Dissolution study of the Mg_2_Si thin films on various degradation conditions. a) Dissolution kinetics and mechanism under temperatures (3 °C, blue; 25 °C, black; 58 °C, red). b) Arrhenius plot of dissolution kinetics under temperatures. c) Dissolution kinetics and mechanism under pH‐controlled solutions (pH 10, blue; pH 7, black; pH 4, red). d) Elemental deconvolution spectra of the Mg 2p (pH 10, 12 h). e) Dissolution kinetics and mechanism under ionic solutions (1 M Na_2_HPO_4_, black; 1 m NaCl, red). f) Elemental deconvolution spectra of the Si 2p (Na_2_HPO_4_, 10 h).

**Figure 3:**
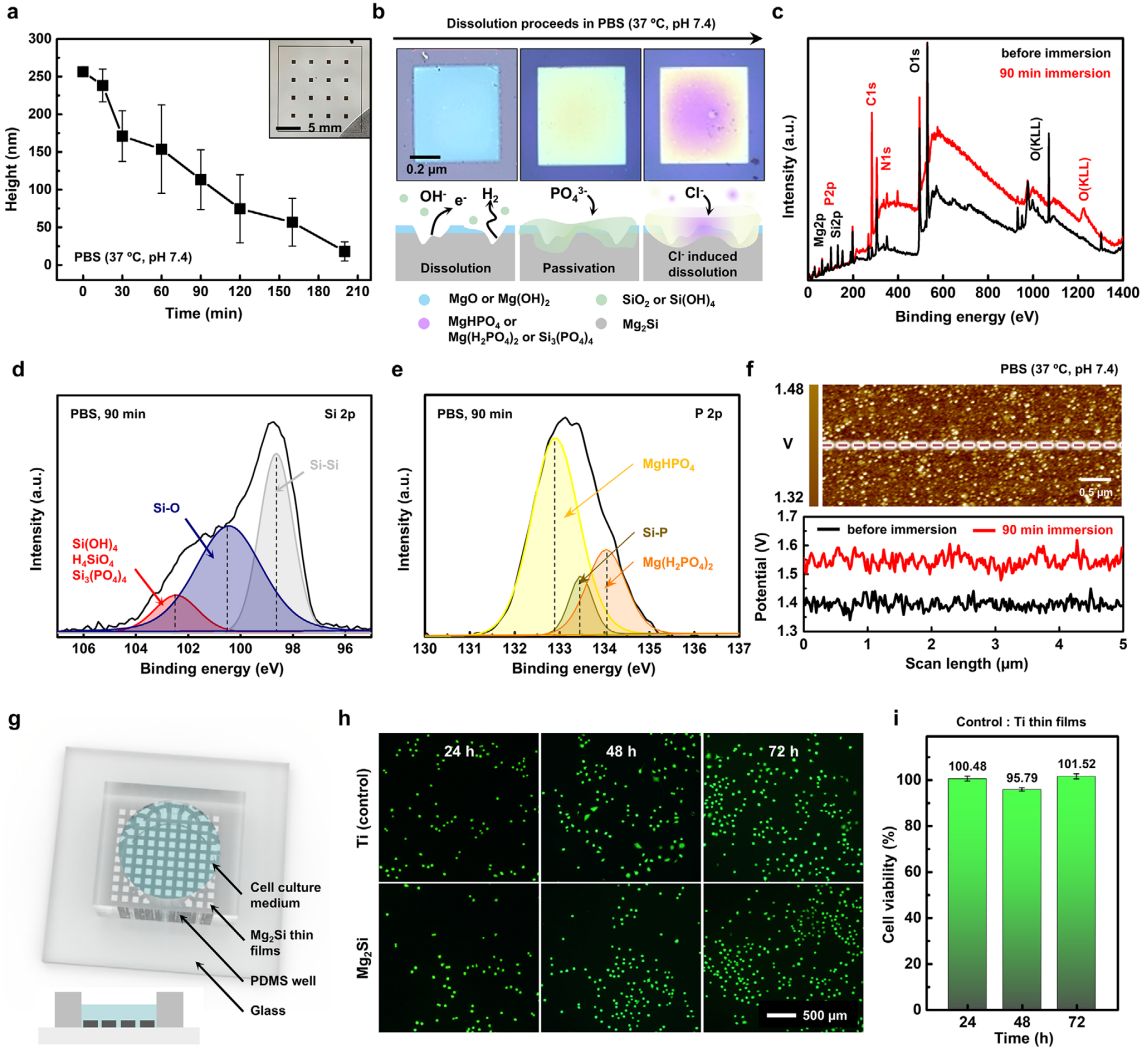
Biodegradation and biocompatibility of the Mg_2_Si thin films. a) Thickness change profiles of the Mg_2_Si thin film patterns (0.7 × 0.7 mm, 250 nm thickness) as a function of immersion time. b) OM images (top row) and schematic diagram (bottom row) of cross‐sectional view depicting the dissolution process of the Mg_2_Si thin films in PBS (37 °C, pH 7.4). c) Wide scan of XPS spectrum of the Mg_2_Si thin films before (black) and after 90 min of PBS immersion (red). High‐resolution deconvoluted spectrum of d) Si 2p and e) P 2p of the Mg_2_Si thin films after 90 min immersion. f) Surface potential mapping of the Mg_2_Si thin films before (black) and after 90 min of PBS immersion (red) at 37 °C (scale bar, 0.5 µm). g) Experiment setup for cell viability test of the Mg_2_Si thin films (20 × 20 µm, 300 nm thickness) using L929 cell (inset, side view of experimental setup). h) Series of fluorescence images containing live/dead cell profiles in Mg_2_Si‐dissolved culture solution (bottom rows) compared to the Ti control group (top row) captured at time intervals of 24, 48, and 72 h after seeding (Scale bar,). i) Cell viability at different culture durations (24, 48, and 72 h).

To demonstrate device‐scale degradation, hydrolysis of Mg_2_Si thin films‐based optoelectronics was examined under a composting environment mimicking high‐moisture biodegradation (58 °C, pH 7.0). Figure 1e shows the time‐sequenced disintegration of a flexible photosensor array fabricated from Mg_2_Si thin films. In this environment, the Mg_2_Si active layers and Mo interconnects, along with the biodegradable polybutylene adipate terephthalate (PBAT) substrate, degrade through hydrolysis. By day 18, the device displays significant thinning, increased transparency, and deformation due to moisture‐induced swelling. By day 30, the structure fragmented, indicating complete disintegration of the device. A more detailed analysis of the dissolution kinetics, interfacial chemistry, and structural degradation under various biologically relevant environments is presented in Figures 2 and 3.

**Figure** **2** systematically investigates the dissolution kinetics and chemical evolution of Mg_2_Si thin films under various aqueous environments by combining thickness measurements and XPS. Mg_2_Si thin film arrays were patterned using photolithography based on the lift‐off technique after negative photoresist (PR) exposure (Figure S6, Supporting Information). Film thickness was measured via surface profilometry across patterned regions, and the average dissolution rate was calculated from the linear reduction in thickness over time. Figure 2a shows the effect of temperature on the hydrolysis of Mg_2_Si thin films in DI water. As the temperature increased from 3 to 58 °C, the dissolution rate rose from 0.20 to 0.70 nm min^−1^. Schematic illustrates the dissolution kinetics as a function of reaction temperature. Elevated temperatures promote thermodynamically accelerated hydrolysis and oxidation processes, leading to the formation of corrosion byproducts such as Mg(OH)_2_ and Si(OH)_4_. The Arrhenius plot in Figure 2b shows a linear relationship between the logarithm of the dissolution rate and the inverse of temperature (T^−1^), yielding an activation energy (E_a_) of 21.63 kJ mol^−1^.

Figure 2c presents the influence of pH on the dissolution behavior using pH buffer solutions (pH 4 and pH 7) and DI water (pH 7) at 37 °C. The dissolution rate was pH‐dependent: 4.58 nm min^−1^ at pH 4, 0.39 nm min^−1^ at pH 7, and 0.17 nm min^−1^ at pH 10. Magnesium readily dissolves in acidic environments via proton‐driven anodic oxidation. This reaction generates substantial amounts of Mg oxides and hydroxides, as confirmed by deconvoluted XPS spectra at pH 4 after 0.5 h immersion (Figure S7, Supporting Information), while its dissolution is suppressed in alkaline media due to the formation of a passivating Mg(OH)_2_ layer.^[^ ^47^ ^]^ In contrast, silicon exhibits limited reactivity under acidic conditions but undergoes accelerated degradation in alkaline environments through hydroxide‐induced cleavage of the Si─O network, yielding soluble silicates.^[^ ^17^ ^]^ Given these opposing pH‐dependent behaviors, the dissolution kinetics of Mg_2_Si appear to be predominantly governed by the Mg component, particularly under physiological or mildly acidic conditions. This trend is supported by XPS analysis of Mg 2p spectra at pH 10 (Figure 2d), which reveals strong signals corresponding to Mg─O (52.0 eV), Mg─(OH) (50.6 eV), Mg(OH)_2_ (49.8 eV), and MgCO_3_ (52.7 eV), indicating the accumulation of mixed oxides and hydroxides on the film surface.^[^ ^48^ ^]^

Figure 2e shows that Mg_2_Si thin films exhibit faster dissolution in 1 m NaCl solution (2.80 nm min^−1^) and the slower dissolution in 1 m Na_2_HPO_4_ solution (0.28 nm min^−1^), compared to DI water (0.39 nm min^−1^) at 37 °C. The accelerated degradation in NaCl is attributed to chloride ions disrupting the surface hydroxide layer, thereby weakening the passivation layer, and initiating pitting corrosion.^[^ ^49^, ^50^ ^]^ Deconvolution spectra of Si 2p in 1 m NaCl solution after 0.5 h of immersion (Figure S8, Supporting Information) reveal the presence of silicon hydroxides and substoichiometric oxides, suggesting that chloride ions accelerate the dissolution of Si‐based surface species through enhanced hydrolysis and oxidative degradation pathways.^[^ ^51^ ^]^ In contrast, phosphate ions in 1 m Na_2_HPO_4_ promote the formation of insoluble phosphate‐based compounds that reinforce the passivation layer and suppress further degradation.^[^ ^52^ ^]^ Figure 2f presents high‐resolution XPS spectra after 10 h of immersion in Na_2_HPO_4_ solution, confirming the distinct effects of phosphate ions on surface chemistry. In phosphate‐rich environments, the Si 2p spectra exhibit peaks corresponding to SiO_2_ (104.1 eV), Si(OH)_4_, H_4_SiO_4_, and Si_3_(PO_4_)_4_ (102.5 eV), indicating the formation of silicate–phosphate complexes within the passivation layer.^[^ ^53^ ^]^ These findings collectively highlight the critical influence of environmental variables—including temperature, pH, and ionic species—on both the rate and mechanism of Mg_2_Si degradation under aqueous conditions.

**Figure** **3** evaluates the biodegradation behavior of annealed Mg_2_Si thin films (350 °C, 12 h) through quantitative dissolution analysis in phosphate‐buffered saline (PBS; 37 °C, pH 7.4) and in vitro cytocompatibility assessment. Figure 3a shows that the linear dissolution rate in PBS is 1.24 nm min^−1^ significantly higher than the rate observed in DI water (0.36 nm min^−1^). This accelerated degradation is attributed to the combined effects of chloride and phosphate ions in PBS. Chloride ions actively penetrate and destabilize surface hydroxide layers, while phosphate ions react with hydrolyzed Mg and Si species to form a chemically complex, yet porous passivation layer composed of oxides, hydroxides, and phosphates.^[^ ^49^, ^50^, ^51^ ^]^ Figure S9 (Supporting Information) presents time‐sequential optical images of patterned Mg_2_Si thin films during dissolution, illustrating the progressive dissolution. Unlike the relatively stable phosphate‐induced passivation observed in Na_2_HPO_4_ solution (Figure 2f), the surface layer formed in PBS remains insufficient to fully suppress corrosion. Figure 3b schematically illustrates the stepwise degradation process of Mg_2_Si thin films in PBS (37 °C, pH 7.4). Upon immersion, hydrolysis and oxidation lead to the formation of MgO, Mg(OH)_2_, SiO_2_, and Si(OH)_4_, accompanied by hydrogen evolution.^[^ ^17^ ^]^ Over time, phosphate ions promote the formation of MgHPO_4_, Mg(H_2_PO_4_)_2_, and Si_3_(PO_4_)_4_, resulting in a heterogeneous passivation layer. However, this layer only partially retards degradation, as chloride ions continue to penetrate and disrupt the surface, leading to the release of byproducts and ongoing dissolution.^[^ ^49^ ^]^ Although the degradation rate in PBS is markedly higher than in DI water, it remains lower than that observed in NaCl solution (1 m), likely due to the relatively low chloride concentration in PBS (0.14 m).

Surface chemical evolution during PBS‐induced degradation was further investigated using XPS. The wide‐scan spectrum (Figure 3c) reveals increased intensities of Mg 2p, Si 2p, P 2p, and O KLL peaks following immersion, indicating the accumulation of corrosion byproducts on the film surface.^[^ ^39^ ^]^ High‐resolution XPS spectrum of Si 2p and P 2p (Figure 3d,e) confirm the presence of Si─O (100.3 eV), Si(OH)_4_, H_4_SiO_4_, and Si_3_(PO_4_)_4_ (102.5 eV), MgHPO_4_ (132.9 eV), Mg(H_2_PO_4_)_2_ (134.0 eV), and Si─P (133.5 eV), suggesting progressive interactions between hydrolyzed Mg/Si species and phosphate ions in the electrolyte.^[^ ^48^, ^50^ ^]^ To visualize the spatial distribution of the passivation layer, surface potential mapping was performed using Kelvin probe force microscopy (KPFM, Figure 3f). After 90 min of PBS immersion, corrosion byproducts—including oxides, hydroxides, and phosphates—were uniformly distributed across the film surface, consistent with a heterogeneous but continuous passivation layer.^[^ ^53^ ^]^ Complementary SEM imaging illustrates the morphological evolution of the Mg_2_Si thin films, revealing increased surface roughness and residue accumulation after immersion (Figure S10, Supporting Information). The presence of these surface residues modulates the local electronic structure and leads to an increase in surface work function, consistent with previous reports on oxide‐induced degradation layers.^[^ ^54^ ^]^

The biocompatibility of Mg_2_Si thin films was assessed by quantifying the viability of L929 fibroblast cells exposed to their dissolution byproducts. A cytotoxicity assay based on cell viability was conducted to evaluate the biological response to the degradation products. Patterned Mg_2_Si dot arrays were fabricated on plasma‐treated SiO_2_ substrates using photolithography with a 10 nm Ti adhesion layer and enclosed within polydimethylsiloxane (PDMS) wells to isolate the films from the surrounding culture medium during in vitro testing (Figure 3g). L929 cells were seeded into the PDMS wells above the patterned films, followed by the addition of culture medium. Cell viability was evaluated by measuring the degree of cell attachment to the Mg_2_Si–patterned glass substrate. Fluorescence imaging of live/dead‐stained cells revealed the spatial distribution of viable cells after 24, 48, and 72 h of incubation (Figure 3h). The relative viability of cells cultured on Mg_2_Si thin films, compared to the Ti dot‐patterned control group, exceeded 101% at 24 h, was 95% at 48 h, and recovered to 101% at 72 h (Figure 3i). The slight reduction in viability observed at 48 h may be attributed to cell detachment, likely caused by limited adhesion between the dot patterns and the glass substrate.

**Figure 4:**
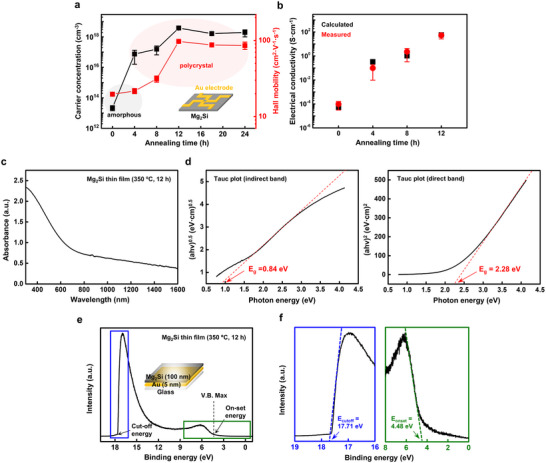
Electronic and optoelectronic properties of the Mg_2_Si thin films. a) Annealing effect on carrier concentration (black) and Hall mobility (blue) of the Mg_2_Si thin films under annealing time variations at 350 °C. b) Calculated (black) and measured electrical conductivities (red) of the Mg_2_Si thin films under annealing time variations at 350 °C. c) Absorbance spectra of the Mg_2_Si thin films (350 °C, 12 h). d) Tauc plot of the Mg_2_Si thin films for indirect and direct bandgap estimation. e) Ultraviolet photoelectron spectroscopy (UPS) profiles of the Mg_2_Si thin films deposited on an interfacial Au layer for work function calibration. f) Enlarged UPS spectrum of cutoff (blue) and onset (green) binding energies for ionization energy and work function estimation.

**Figure** **4**a presents the results of Hall effect measurements showing carrier concentration and mobility as functions of annealing duration at a fixed temperature of 350 °C. These results support a clear correlation between thermal processing and electronic transport behavior. The as‐deposited amorphous Mg─Si exhibited a carrier concentration of 2.23 × 10^13^ cm^−3^ which increased to 7.46 × 10^16^ cm^−3^ after 4 h and 1.58 × 10^17^ cm^−3^ after 8 h of annealing, reaching a maximum of 3.80 × 10^18^ cm^−3^ at 12 h; these results indicate n‐type conductivity of the Mg_2_Si. Hall mobility increased from an initial value of 19.63 cm^2^ V^−1^ s^−1^ to a maximum value of 97.13 cm^2^ V^−1^ s^−1^ at 12 h, comparable to polycrystalline Si (10–200 cm^2^ V^−1^ s^−1^) at similar doping levels (10^18^–10^19^ cm^−3^).^[^ ^55^, ^56^ ^]^ The increases in carrier concentration and mobility with annealing duration are attributed to the crystallization into polycrystalline Mg_2_Si, accompanied by the formation of Mg–Si bonds.^[^ ^55^ ^]^ Beyond the optimized annealing temperature and duration, the carrier concentration and Hall mobility exhibit a saturation trend at ≈1.97 × 10^18^ cm^−3^ and 85.72 cm^2^ V^−1^ s^−1^

Figure 4b presents the electrical conductivity of Mg_2_Si thin films as a function of annealing duration at 350 °C. The electrical conductivity was estimated using the relation σ = e(nµ_e_ + pµ_p_), where n and p are the carrier concentrations and µ_e_, µ_p_ their respective mobilities obtained from Hall measurements in Figure 4a. Both calculated and measured conductivities increased consistently with annealing time. The measured conductivity rose from ≈9.8 × 10^−5^ S cm^−1^ for the as‐deposited film to ≈80 S cm^−1^ after 12 h of annealing, while the calculated values followed a similar trend, increasing from 0.91 to 82 S cm^−1^ The close agreement between the two data sets confirms that charge transport is primarily governed by electrons, with minimal hole contribution. This behavior is likely associated with the intrinsically low hole density in n‐type Mg_2_Si thin films, possibly arising from self‐doping effects or native defect states.^[^ ^57^, ^58^ ^]^ Complementary time‐domain thermoreflectance (TDTR) measurements confirm the thermal conductivity of the Mg_2_Si thin films. Figure S11 (Supporting Information) presents TDTR signals, the ratio of in‐phase to out‐of‐phase voltage, as a function of time delay, where the annealed Mg_2_Si thin films (350 °C, 12 h) exhibit a higher ratio signal compared to the as‐deposited amorphous Mg─Si mixture. This increase in ratio signal corresponds to an enhancement in thermal conductivity from 1.1 to 1.6 W m^−1^ K^−1^ which is attributed to the crystallization of Mg2Si thin films (Note S3, Supporting Information).

The optoelectronic properties of Mg_2_Si thin films were evaluated using UV–vis absorbance and ultraviolet photoelectron spectrum. Figure 4c shows the UV–vis absorbance spectrum of a polycrystalline Mg_2_Si thin film (≈100 nm thickness), which displays a strong band‐to‐band transition near 300 nm, along with broad absorption spanning the near‐UV to visible range. The absence of sharp spectral features and a gradual decay toward the NIR region suggest contributions from defect states and surface oxides generated during deposition and annealing.^[^ ^59^, ^60^ ^]^ In the long‐wavelength region, the extended absorption tail is attributed to indirect transitions and free carrier absorption, consistent with its degenerate n‐type behavior of Mg_2_Si.^[^ ^61^ ^]^ The optical bandgap was determined by plotting Kubelka–Munk‐transformed absorbance spectra against photon energy. Tauc plots for indirect and direct transitions yielded bandgap energies of 0.84 and 2.28 eV, respectively (Figure 4d). The coexistence of indirect and direct bandgap transitions indicates broadband optical activity, supporting the applicability of Mg_2_Si in bioresorbable optoelectronic and thermoelectric systems.

Ultraviolet photoelectron spectroscopy (UPS) was employed to analyze the surface electronic structure of the Mg_2_Si thin films annealed at 350 °C for 12 h. This technique enabled probing of the valence band edge and determination of the work function, probing the electronic alignment at the vacuum interface (Figure 4e). As shown in Figure 4f, the magnified UPS spectrum reveals a secondary electron cutoff at 17.71 eV and a valence band onset at 4.48 eV.^[^ ^62^ ^]^ The first ionization energy, calculated as the sum of these values, is ≈7.97 eV, while the work function is determined to be 3.49 eV. Compared to representative biodegradable inorganic semiconductors—such as polycrystalline Si (8.15, 4.61 eV), ZnO (7.73, 5.31 eV), amorphous IGZO (7.20, 4.30 eV), and Ge (7.89, 4.76 eV)—Mg_2_Si exhibits slightly lower ionization energy and work function. This result suggests a higher density of occupied surface states near the Fermi level, potentially arising from native oxides and atomic defects on the surface.^[^ ^57^, ^59^, ^60^ ^]^

**Figure 5:**
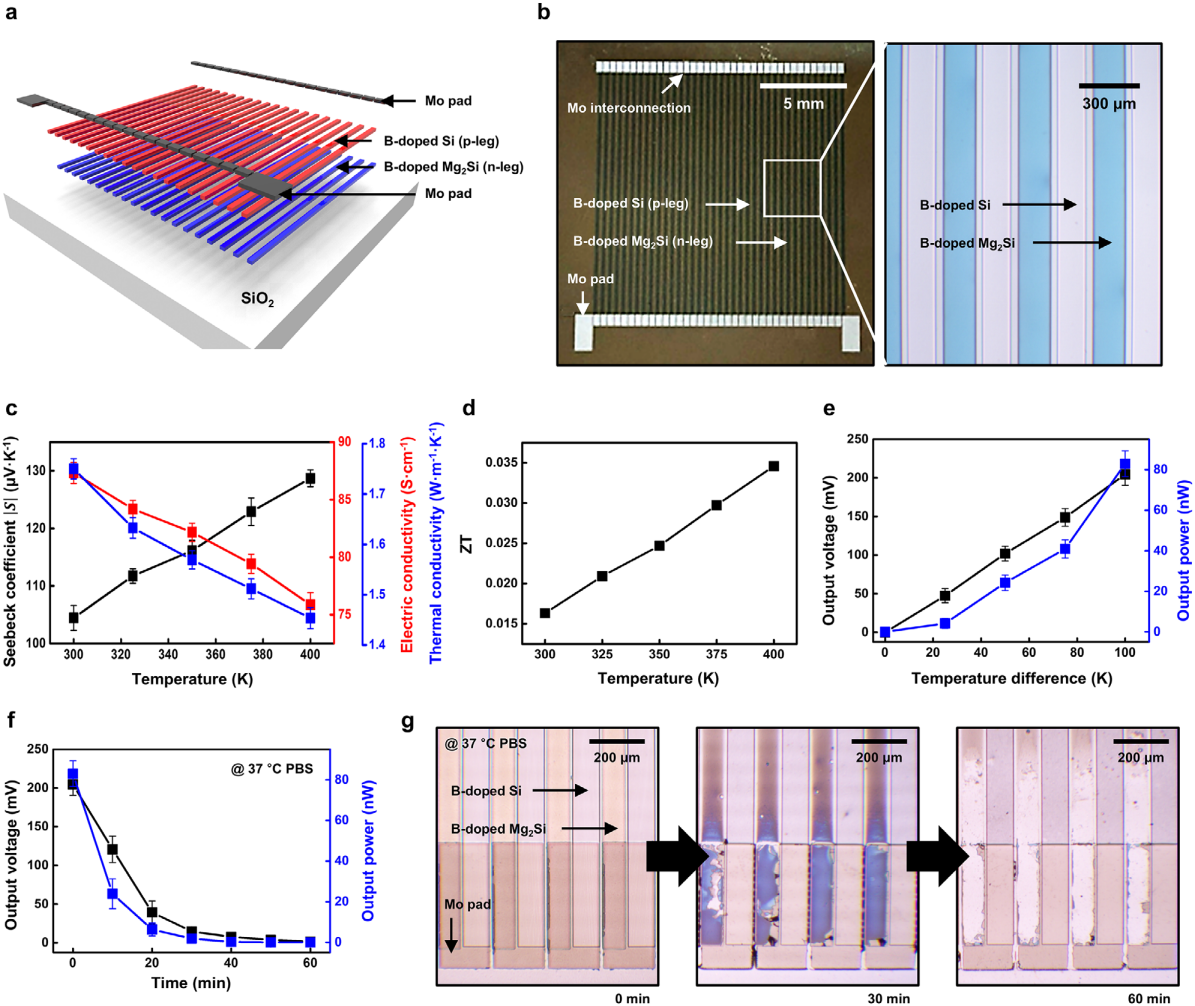
Thermoelectric properties of the B‐doped Mg_2_Si thin films and the performance of the thermoelectric generator (TEG) based on them. a) Schematic illustration and b) photographic image of the TEG device fabricated using the B‐doped Mg_2_Si thin film, showing the entire device (left) and a magnified view of an individual element (right). c) Temperature‐dependent Seebeck coefficient (black), electrical conductivity (red), and thermal conductivity (blue) of the B‐doped Mg_2_Si thin film. d) Calculated figure of merit (ZT) values of the B‐doped Mg_2_Si thin films as a function of temperature. e) Output voltage (black) and output power (blue) of the TEG measured under various temperature gradients. f) Decrease in output voltage (black) and output power (blue) during PBS degradation at 37 °C. g) Sequential optical images illustrating the degradation of the B‐doped Mg_2_Si thin film elements in PBS at 37 °C. Error bars represent standard deviation (small and thus may be invisible at later time points).

Mg_2_Si possesses potential for biodegradable thermoelectric generator (TEG) applications due to its unique combination of tunable, sufficiently high electrical conductivity and Seebeck coefficient, intrinsically low thermal conductivity, and favorable dissolution properties.^[^ ^63^, ^64^, ^65^, ^66^, ^67^, ^68^, ^69^, ^70^, ^71^ ^]^ Previous Mg_2_Si‐based TEG studies have achieved higher figure of merit (ZT) values through high‐temperature sintering; however, such approaches are generally less compatible with biodegradable device fabrication processes that involve polymer substrates (Table S1, Supporting Information). In contrast, our work demonstrates that measurable thermoelectric output can be achieved using only a low temperature annealing process, ensuring process compatibility with flexible and biodegradable systems. Mg_2_Si thin film exhibits lower electrical conductivity compared to the bulk form, but its reduced thermal conductivity offers effective thermoelectric operation. **Figure** **5** presents a TEG fabricated with B‐doped Mg_2_Si thin films. Figure 5a,b show the schematic layout and optical image of the fabricated TEG, respectively. The TEG consists of 60 thermocouple pairs (B‐doped Mg_2_Si, ≈200 nm thickness, n‐type legs; B‐doped silicon, ≈200 nm thickness, p‐type legs) and interconnects (molybdenum, ≈400 nm thickness). Besides Mg_2_Si, Si, and Mo are likewise known to exhibit favorable biodegradation characteristics.^[^ ^1^, ^7^, ^20^, ^72^ ^]^ Detailed dimensional parameters and fabrication procedures are provided in Figures S12 and S13 (Supporting Information). The device was fabricated on a thermally insulating silicon oxide layer to suppress parasitic heat leakage. The thin film geometry reduced lateral thermal conduction, while vertically elongated thermoelectric legs facilitated a well‐defined temperature gradient across the device.^[^ ^73^, ^74^, ^75^ ^]^

Figure 5c shows the temperature‐dependent variations in the Seebeck coefficient, electrical conductivity, and thermal conductivity of B‐doped Mg_2_Si thin films. While annealing enhanced electrical properties, the inherently small grain size of polycrystalline Mg_2_Si reduced carrier mobility,^[^ ^76^, ^77^, ^78^ ^]^ resulting in lower electrical conductivity than sintered Mg_2_Si and limiting its direct applicability in TEGs. Boron doping (molar ratio ≈0.7) was introduced to enhance electrical conductivity (≈19–88 S cm^−1^ at 300 K) by increasing the electron carrier concentration of Mg_2_Si (Figure S14, Supporting Information). B‐doped Mg_2_Si exhibited n‐type semiconducting behavior, with boron atoms substituting at magnesium sites and donating excess electrons.^[^ ^79^ ^]^ With increasing temperature, the absolute value of the Seebeck coefficient increased, while both the electrical and thermal conductivities decreased—consistent with trends reported for sintered Mg_2_Si.^[^ ^65^, ^67^, ^69^ ^]^ The Seebeck coefficient of the polycrystalline Mg_2_Si was comparable to that of sintered B‐doped Mg_2_Si with microscale grains, whereas the lower electrical conductivity was due to reduced carrier mobility, and the lower thermal conductivity resulted from enhanced phonon‐phonon scattering with increasing temperature. Figure 5d presents the figure of merit (ZT) calculated based on the measured thermoelectric parameters. A maximum ZT value of ≈0.0357 was achieved at elevated temperatures. Although the ZT value is lower than that of sintered B‐doped Mg_2_Si, which typically exhibits a ZT of ≈0.2, it surpasses those of nanostructured silicon,^[^ ^80^ ^]^ primarily due to the ultralow thermal conductivity of the polycrystalline Mg_2_Si, which effectively limits phonon‐mediated heat transport.

The output characteristics of the TEG device based on B‐doped Mg_2_Si thin films demonstrate stable voltage generation and power output comparable to Si‐based counterparts (Figure 5e). A maximum output voltage of 202.53 mV and power of 82.32 nW were achieved under a temperature difference of 100 K, corresponding to a calculated power output density of 0.338 µW cm^−2^ K^−2^, thereby demonstrating the viability of the B‐doped Mg_2_Si thin film for energy harvesting applications.

Electrical properties of biodegradable electronics inherently degrade as their constituent components progressively decompose in aqueous environments.^[^ ^1^, ^7^, ^20^ ^]^ Fully biodegradable TEG showed a rapid performance decline within 30 min when immersed in PBS (pH 7.4, 37 °C) (Figure 5f). This behavior is attributed to the rapid degradation of B‐doped Mg_2_Si, as sequential degradation images clearly show that B‐doped Mg_2_Si dissolves significantly faster than the other components (Figure 5g). As degradation progressed, the Seebeck coefficient decreased, resulting in a gradual loss of output voltage and a marked increase in internal resistance. Consequently, the output power dropped more significantly. Subsequent OM images of material degradation confirmed the degradation of other components, verifying the complete device dissolution (Figure S15, Supporting Information). The use of polybutylene adipate terephthalate (PBAT) encapsulation on the degradation process can prolong the performance duration (Figure S16, Supporting Information). The B‐doped Mg_2_Si thin film‐based biodegradable TEGs present distinct advantages among transient energy device platforms by continuously harvesting power from ambient thermal gradients without dependence on external stimuli or finite electrochemical reactants. This intrinsic capability enables reliable, maintenance‐free operation in conditions where optical access, mechanical excitation, or battery replacement are impractical for photovoltaic/piezoelectric devices, and batteries. Accordingly, our TEG‐based energy harvesting demonstration establishes a viable route toward sustainable, self‐sufficient, and fully transient electronic systems.

**Figure 6:**
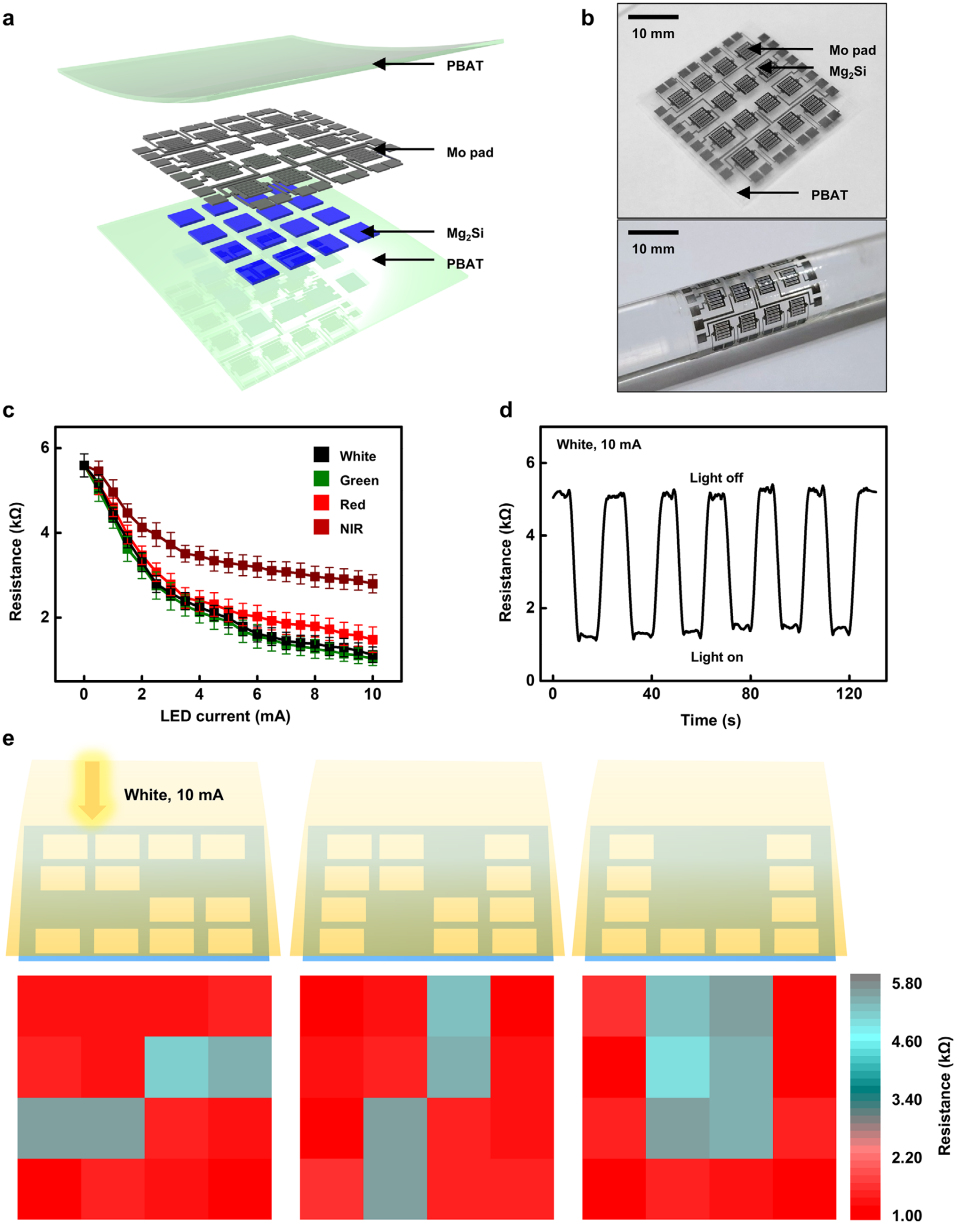
Performance of a photo‐resistive optoelectronic device based on polycrystalline Mg_2_Si thin film. a) Schematic illustration and b) photographic images of the Mg_2_Si thin film‐based photosensor, showing the entire device (up) and its conformal contact to the curved surface (down). c) LED current–dependent resistance change under white (400–800 nm, black), green (527 nm, green), red (630 nm, red), and NIR (940 nm, wine) light. d) On/off switching behavior of the photosensor under white LED at 10 mA of LED current. e) Photo‐imaging of the Mg_2_Si thin film‐based photosensor array displaying the letters “S”, “N”, and “U”.

Mg_2_Si exhibits photoresponsive behavior owing to its intrinsic bandgap characteristics.^[^ ^46^, ^81^, ^82^ ^]^ Compared to previous biodegradable semiconductors, Mg_2_Si possesses a low indirect bandgap energy of ≈0.84 eV, enabling efficient sensing of relatively longer wavelengths.^[^ ^1^, ^14^, ^81^, ^82^, ^83^, ^84^, ^85^ ^]^ While most Mg_2_Si‐based NIR photosensors have been realized in photodiode configurations, in this work, we adopted a light‐dependent resistor (LDR) design to ensure process compatibility with biodegradable soft substrates and to highlight mechanical flexibility. **Figure** **6** illustrates the application of a biodegradable photosensor device fabricated using Mg_2_Si thin films. Figure 6a,b show a schematic layout and an optical image of the device, respectively. The stack comprises an Mg_2_Si active layer and serpentine Mo pads on a flexible PBAT substrate, with a PBAT encapsulation layer to protect the device from mechanical deformation and environmental degradation. Notably, PBAT, used for the substrate and encapsulation layer, is also known to possess excellent biodegradability, in addition to the aforementioned materials.^[^ ^86^, ^87^ ^]^ The serpentine geometry of Mo pads increases the effective electrode–active‐layer contact area, thereby enhancing the photoresponse sensitivity.^[^ ^88^ ^]^ The device is configured as a 4 × 4 array to allow image mapping without inter‐element interruption; detailed dimensions and fabrication procedures are provided in Figures S17 and S18 (Supporting Information). The entire device is flexible and forms conformal contact on curved surfaces owing to the inherent flexibility of PBAT used for both substrate and encapsulation,^[^ ^89^, ^90^ ^]^ and the mechanical compliance of thin film Mo and Mg_2_Si (Figure 6c).^[^ ^91^ ^]^

Figure 6d summarizes the photo‐sensing characteristics as a function of wavelength and light intensity. The increase of light intensity leads to a monotonic decrease in resistance for all wavelengths due to an increased density of photo‐generated carriers, demonstrating reliable light intensity sensing across the tested spectral range. Consistent with the result in Figure 2e, where absorbance increases with decreasing wavelength, it is observed that the resistance decreases more significantly at lower wavelengths. The resistance drops sharply at low light intensities and then tapers off at higher intensities across all wavelengths, consistent with saturation of carrier generation by optical absorption, which limits further resistance reduction.^[^ ^92^ ^]^ A finite response is observed in the NIR because Mg_2_Si possesses a low indirect bandgap of ≈0.84 eV; however, the thin film form of Mg_2_Si (200 nm thickness) disfavors absorption of long‐wavelength photons with long penetration depth, resulting in a lower NIR sensitivity than that in the visible region.^[^ ^93^ ^]^ In particular, when the thickness of the Mg_2_Si thin films was increased to 1 µm, the photosensitivity at 940 nm approximately doubled from 2.00 to 3.96, indicating enhanced photoresponse performance with a response time of 1.88 s and a retention of 97.6% after 10 on/off cycles (Figure S19, Supporting Information). Moreover, under this condition, extended photoresponsivity was observed at longer wavelengths ≈1300 nm (Figure S20, Supporting Information). In the visible range, the resistance under illumination at 10 mA decreases to below one‐fifth of the dark value, reflecting strong direct transitions near the high direct bandgap. Figure 6e shows the on/off switching of the photosensor under a white light‐emitting diode (LED) at 10 mA, where rapid resistance modulation indicates a short response time. Repeated cycling produces negligible drift in the illuminated resistance, highlighting the operational stability of the optoelectronic device. Image mapping of photosensor array was obtained by using the same white LED (10 mA). Using a black‐paper mask, only the illuminated pixels show pronounced resistance drops, yielding high selectivity with minimal interpixel interruption and enabling reconstruction of target patterns (Figure S21, Supporting Information). To demonstrate device robustness in mechanical deformation, we verified the photoresponse of the flexibly configured Mg_2_Si thin film‐based optoelectronic device after repeated bending cycles of 10 000. Samples were prepared with a transfer process of the Mg_2_Si thin film (200 nm thickness) onto a PBAT substrate in a rod‐shaped geometry (4 × 0.25 cm). The sample was subjected to bending cycles of 5000 with a bending radius of 2 cm. After the bending test, the initial resistance increased moderately from 10.13 to 13.24 kΩ, while the photosensitivity under white‐light illumination changed only slightly from 4.55 to 4.47, indicating that the device retained sufficient functionality as a photoresistor even after extensive mechanical deformation (Figure S22, Supporting Information). These results underscore the suitability of biodegradable polycrystalline Mg_2_Si for transient image‐sensor applications; further miniaturization of individual sensing elements and array expansion should improve spatial resolution. Overall, the Mg_2_Si‐based photosensor presented in this work demonstrates a promising route toward an eco‐friendly transient optoelectronic platform that simultaneously achieves mechanical flexibility, stable photoresponse, and complete biodegradability (Figure S23, Supporting Information).

## 3 Conclusion

In conclusion, this study introduces magnesium silicide (Mg_2_Si) as a promising candidate for narrow‐bandgap, biodegradable inorganic semiconductor thin films suitable for transient electronic systems. Polycrystalline Mg_2_Si thin films were synthesized through optimized conditions for sputtering and thermal annealing processes. The films exhibited hydrolysis behavior in both ionic solutions and PBS environments, with a dissolution rate analysis and byproduct characterization confirming safe and predictable biodegradation. With an indirect bandgap energy of ≈0.84 eV, the polycrystalline Mg_2_Si thin films demonstrated n‐type electrical conductivity and thermal conductivity. Leveraging these electrical and thermal properties, thermoelectric generators and photosensors were fabricated to validate ist integration feasibility at the device scale. The confirmed functional operation, together with the demonstrated compatibility of the Mg_2_Si thin films with standard photolithographic patterning, highlights its potential as a narrow‐bandgap, biodegradable semiconductor for use across a broad range of transient electronic applications. Overall, Mg_2_Si offers strong potential to replace conventional wide‐bandgap materials, particularly for advancing the functional capabilities of biodegradable electronics in energy‐harvesting and broadband optoelectronic systems. Although our system demonstrates robust performance, its ability to convert ubiquitous thermal gradients into stable electrical output further underscores its promise as a core material platform for developing self‐sustaining and transient electronic systems.

## 4 Experimental Section

### Fabrication of Polycrystalline Mg_2_Si Thin Films

Biodegradable Mg_2_Si thin films were prepared by using a magnetron sputtering system (J Vacuum Technology, Republic of Korea). The chamber was evacuated to a base pressure below 5 × 10^−6^ Torr. The films were deposited at a working pressure of 5 mTorr, RF deposition power of 100 W for Mg_2_Si and B‐doped Mg_2_Si (99.99%, 2‐inch diameter, 0.5‐inch thickness, KRT Metals, Republic of Korea), and deposition duration of 10–30 min with the substrate holder rotation of 4 rpm for film homogeneity. For the polycrystalline Mg_2_Si, the thin films were annealed in a thermal chemical vapor deposition (Thermal CVD, ScenTech, Republic of Korea) at 250–350 °C under a constant Ar flow of 3 sccm for 4–24 h.

### Crystallographic Analysis of Mg_2_Si Thin Films

Grazing incident X‐ray diffraction (GI‐XRD) patterns were obtained using an XRD diffractometer (D8 Advance, Bruker, USA) with a CuKα radiation across an angular range of 20–80° in 0.02° increments to ascertain the crystal orientation of the Mg_2_Si thin films. The crystal microstructures and phases were confirmed using spherical aberration–corrected scanning transmission electron microscopy (CSTEM; Themis Z, Thermo Fisher Scientific Inc., USA) to obtain high‐angle annular dark‐field (HAADF) imaging in scanning TEM (STEM) mode. Furthermore, lamina sample preparation of the Mg_2_Si thin films deposited on the Si(100) was prepared and observed by using focused‐ion beam (FIB; Helios 5 UC, FEI Inc., USA). Grain size determination was manually conducted from the high‐resolution transmission microscopy (HRTEM) images by dividing the number of intersections by the actual line length in the HRTEM image (grain size average = 1/number of intersections/actual length of the line). The surface morphology and potential distribution were observed by scanning electron microscopy (SEM; MERLIN Compact, ZEISS, Germany) and atomic force microscopy (AFM; NX‐10, Park Systems, Republic of Korea).

### Biodegradation Test in Compost

The compost used for the biodegradation test of the Mg_2_Si‐based photosensor array consisted of 25 wt.% of swine manure, 15 wt.% of chicken manure, 15 wt.% of sawdust, 20 wt.% of natural leaf mold, 18 wt.% of coco peat, 4 wt.% of zeolite, and 3 wt.% of soil microorganisms. The moisture content was adjusted by mixing the compost with deionized water at a 1:1 weight ratio after drying to achieve a 100% water‐holding capacity. The biodegradation process was conducted under an oxygen‐supplied aerobic environment to ensure sufficient microbial activity during composting. All experimental procedures for the composting test were performed in compliance with the national standard ISO 14855‐1.

### Optical Properties of Mg_2_Si Thin Films

Absorbance spectrum of the Mg_2_Si thin films was obtained using UV–vis spectroscopy (LAMBDA 1050+, PERKIN ELMER, USA), ranging from 300 to 1600 nm. The elemental compositions of the Mg_2_Si thin films were analyzed by XPS (VersaProbe III, ULVAC‐PHI Inc., Japan) conducted using a monochromatic X‐ray source induced by 12 kV and 18 mA Al Kα radiation on the Mg_2_Si thin films. After Ar^+^ sputtering to a depth of 5–20 nm from the surface, the oxygen content was found to be 2–5 at%. The relative contents of the alloy elements were calculated using the deconvolution of the obtained XPS peaks. Raman spectroscopy (LabRAM HR Evolution, HORIBA, Japan) was used to acquire the Raman spectrum of the as‐deposited Mg─Si and annealed Mg_2_Si thin films on different vibration modes. Ultraviolet photoelectron spectroscopy (UPS) measurements were conducted in an ultrahigh vacuum system (VersaProbe III) with an operating pressure of 4  ×  10^−9^ Torr. UV light was generated using lamp operated at 5  ×  10^−5^ Torr and 1 A emission to maximize He I radiation at 21.2 eV. The He I:He II intensity ratio was ≈5000:1. The sample normal was oriented toward the electron detector. Work functions were determined by identifying the secondary electron cut‐off (SECO) energy in UPS. This was identical to measuring the photoemission total band width with the sample grounded and Fermi energy fixed to 21.2 eV kinetic energy (0 eV binding energy). The Fermi energy position was calibrated with a sputtered Au sample.

### Tauc Plotting

Tauc conversion of the absorbance spectrum of the Mg_2_Si thin films was conducted by using the following equation:

(1) $${\left(\alpha v\right)}^{1/n}=A\left(hv-E_{g}\right)$$

where h is plank's constant, *ν* is the photon's frequency, α is the absorption coefficient, E_g_ is the band gap, and A is a proportionally constant. The value of the exponent denotes the nature of the electronic transition, whether allowed or forbidden, and whether direct or indirect (n = 1/2 for direct transition, n = 2 for indirect transition).

### Electronic Properties of Mg_2_Si Thin Films

Carrier concentration and Hall mobility were investigated using a Van der Pauw Hall measurement instrument (HL5500PC, Nanometrics, Canada) under a magnetic field up to 500 mT with a 20 mV target current. n and µ were determined by 𝑛 = 1⁄𝑒𝑅 and 𝜇 = 𝜎𝑅, respectively.

### Cell Culture

L929 cells were cultured in Dulbecco's Modified Eagle Medium (DMEM; Welgene, Republic of Korea) supplemented with 10% fetal bovine serum (FBS; VWR International, USA) and 1% penicillin/streptomycin (Gibco, USA). The cells were maintained in a humidified cell incubator at 37 °C and 5% CO_2_ atmosphere. Cells were detached at ≈80% confluence from the culture dish using a 0.05% trypsin‐EDTA solution (Gibco) and seeded onto samples at a density of 5 x 10^3^ cells cm^−2^

### Cell Viability

The viability of L929 was evaluated by Cell Counting Kit‐8 (CCK‐8; Dojindo, Japan) and Live/Dead staining (Invitrogen, USA) at 24, 48, and 72 h. For the CCK‐8 assay, 10% CCK‐8 solution was added to each well, followed by a 2 h incubation. Absorbance was measured at 450 nm using a microplate reader (Promega, USA). The Live/Dead assay was performed using Calcein AM and Ethidium homodimer‐1 to stain live and dead cells, respectively. A staining solution was prepared by adding 2 µL of the Calcein AM and 8 µL of the Ethidium homodimer to 4 mL of Dulbecco's phosphate‐buffered saline (DPBS; Welgene). The solution was added to the cell‐seeded samples and incubated for 30 min at room temperature. Stained cells were then imaged using a fluorescence microscope (IX71, Olympus, Japan) with dead cells stained in red and live cells stained in green.

### Thermoelectric Property Measurements

The Seebeck coefficient was determined by applying a temperature gradient across the thin film and measuring the resulting voltage using a Keithley SourceMeter (Model 2612B, USA). The Seebeck coefficient was then calculated based on the temperature‐dependent Seebeck voltage. Electrical conductivity was measured using the standard four‐probe method (M4P302, MStech, Republic of Korea) with a Keithley SourceMeter (Model 2450). The thermal conductivity was calculated using the following equation:

(2) $$k=\rho C_{p}\alpha$$

where ρ is the material density, C_p_ is the specific heat capacity, and α is the thermal diffusivity. In this study, the density of Mg_2_Si was measured using a spectroscopic ellipsometer (M‐2000DI, J.A. Woollam Co., Inc., USA), the heat capacity was measured using a differential scanning calorimeter (Discovery DSC 25, TA Instruments, USA), and the thermal diffusivity was obtained using a laser flash analyzer (LFA 467, NETZSCH, Germany).

### Fabrication of the Thermoelectric Device and Thermoelectric Characterization

The thermoelectric device was fabricated following the process illustrated in Figure S13 (Supporting Information). To suppress heat conduction into the substrate, oxide wafers consisting of Si with a ≈1 µm SiO_2_ layer (KRTLAB, Republic of Korea) were used. Amorphous silicon was first deposited onto the wafer via sputtering (J Vacuum Technology), followed by spin‐coating of a boron dopant solution (Filmtronics, USA). Doping was carried out via thermal diffusion at 1050 °C. The resulting thermal oxide was subsequently removed using HF (SAMCHUN, Republic of Korea) and nano‐strip solution (CMCMaterials, USA). To define the p‐leg regions, AZ5214 positive photoresist (Merck Performance Materials, Germany) was patterned using an aligner (MDA‐400S, MIDAS, Republic of Korea), followed by isolation etching with a reactive ion etcher (JV19RIE‐8AP, J Vacuum Technology, Republic of Korea) using SF_6_ gas. The n‐leg regions were then patterned using AZ2070 negative photoresist (Merck Performance Materials, Germany) and the same aligner. B‐doped Mg_2_Si was deposited via sputtering, and lift‐off was performed in acetone. The deposited Mg_2_Si was annealed at 350 °C for 12 h. Subsequently, the metal pads and interconnects were defined by repeating the photolithography process using AZ2070 negative photoresist and the aligner. Molybdenum was deposited by sputtering and patterned using lift‐off to complete the fabrication of the thermoelectric device. The thermoelectric performance was measured using a Keithley SourceMeter (Model 2450) under a defined temperature gradient generated by a heat source. For each temperature condition, the performance was evaluated using three different samples. The degradation tests were conducted in PBS solution at 37 °CC, also using three independent samples.

### Fabrication of the Optoelectronic Device and Optoelectronic Characterization

The device was fabricated as illustrated in Figure S18 (Supporting Information). A polyimide (poly(pyromellitic dianhydride‐co‐4,4′‐oxydianiline), Sigma–Aldrich, USA) was spin‐coated onto Si wafers and thermally cured at 150 °C for 5 min followed by 220 °C for 1 h. Mg_2_Si element regions were defined by photolithography using AZ2070 negative photoresist and an aligner. Mg_2_Si was deposited by sputtering, and lift‐off was performed in acetone. The deposited Mg_2_Si was then annealed at 350 °C for 12 h. Subsequently, metal pads and interconnects were patterned by repeating the photolithography steps with AZ2070 and the same mask aligner; molybdenum (Mo) was deposited by sputtering and patterned by lift‐off. For release and transfer, the entire device on the polyimide was retrieved using polydimethylsiloxane (PDMS) stamping. The polyimide layer on the PDMS stamp was removed by O_2_ reactive ion etching. The released device was transfer‐printed onto a polybutylene adipate terephthalate (PBAT) film preheated to 110 °C, and a PBAT encapsulation layer was spin‐coated to complete the device. Device performance was evaluated inside a light‐tight enclosure lined with black paper to block ambient illumination. Photocurrent was recorded using a Keithley SourceMeter (Model 2450) under light‐emitting diode (LED; infrared; XTNI12BF, SunLED, USA; red, WP7113LSURDK, Kingbright, USA; green and white, LKP0585, LK Embedded Lab, Republic of Korea) illumination while varying the emission wavelength and drive current. The photocurrent measurements were performed for each emission wavelength and drive current condition, using three distinct samples to ensure reproducibility. On/off switching characteristics were obtained by modulating the LED. For imaging/mapping measurements, a black paper mask was placed on the device surface, and the array response under patterned illumination was acquired.

## Conflict of Interest

The authors declare no conflict of interest.

## Supporting information



Supporting Information

## Data Availability

The data that support the findings of this study are available from the corresponding author upon reasonable request.
