# Supplementary material for: Dissolution Study of Biodegradable Magnesium Silicide Thin Films for Transient Electronic Applications
Source: Adv Sci (Weinh). 2025 Nov 25;13(15):e18093. doi: 10.1002/advs.202518093 (PMC13042761; doi:10.1002/advs.202518093)
Supplement: Supplementary file 1 — Supporting Information [file ADVS-13-e18093-s001.docx]

Supporting Information

Dissolution Study of Biodegradable Magnesium Silicide Thin Films for Transient Electronic Applications

Ji-Woo Gu, Jun-Seok Shim, Minjung Chae, Yoonseong Jung, Su-Min Kim, Young-In Ryu, Jae-Hwan Lee, Sung-Woo Kim, Kyung-Sub Kim, Tae-Woo Lee, Edyta Wyszkowska, Jungho Shin, Hyejin Jang, Ju-Young Kim, Myoung-Ryul Ok, Jong-hyoung Kim*, Jae-Young Bae*, Seung-Kyun Kang*

**Note S1. Crystallite size approximation**

Crystalline size and crystallinity approximation of thin films were calculated by using the following Scherrer equation:^[1]^

$\beta\cos\theta=K\lambda/D+\varepsilon\cos\theta$ (1)

where D is the crystallite size, K is the Scherrer constant whose value is ~ 0.9, λ is a wavelength of the incident X-ray radiation used, θ is the Bragg angle, β is the full width at half maximum (FWHM) value, and ε is the lattice strain constant. The crystallite sizes were drawn with the help of the first three peaks for each diffractogram.

**Note S2. Nanoindentation test**

Nanoindentation was carried out using a MEMS-based indenter (FT-I04, Oxford Instruments) operated in continuous stiffness measurement (CSM) mode at 150 Hz. True displacement control was maintained by the integrated MEMS load cell and long-range piezo actuator, with indentations performed at 20 nm/s under a 3 nm CSM oscillation to a maximum depth of 150 nm. The CSM signal revealed a hardness plateau at 25–30 nm, taken as the thin film response to minimize surface and substrate effects. Large-area mapping over a 100 × 100 µm^2^ region with 5 µm spacing yielded 400 indents, providing statistically robust property distributions.

**Note S3. Time domain thermoreflectance (TDTR) measurement**

Measurements were conducted using a pump-probe optical setup based on an fs pulsed laser.^[2,3]^ A Ti:Sapphire laser emitted pulses at an 80 MHz repetition rate, with a 780 nm center wavelength. A polarizing beam splitter (PBS) separated the beam into pump and probe components. The pump beam, modulated at 10.9 MHz by an electro-optic modulator (EOM), acted as a periodic heater. The probe beam, time-delayed compared to the pump beam via a linear delay stage, detected the surface temperature change via thermoreflectance. Specimens were prepared as follows: 1) Mg_2_Si film was deposited on bare Si substrate using DC sputtering. 2) The deposited films were annealed at 350 ℃ for 0, 4, 8, 12, and 24 hours, respectively. Each specimen was coated with an Al thin film of about 80 nm thickness, which served as a transducer due to its large thermoreflectance coefficient. The reflected probe beam was detected by a Si photodiode, and the 10.9 MHz frequency signal was extracted using a lock-in amplifier. TDTR measurements were conducted on three different spots for each sample.

References

[1] A. T. D’Agostino, *Analytica Chimica Acta* 1992, **262**, 269-275

[2] D. G. Cahill, *Rev. Sci. Instrum.* 2004, **75**, 5119–5122.

[3] K. Kang, Y. K. Koh, C. Chiritescu, X. Zheng, D. G. Cahill, *Rev. Sci. Instrum.* 2008, **79**, 114901.


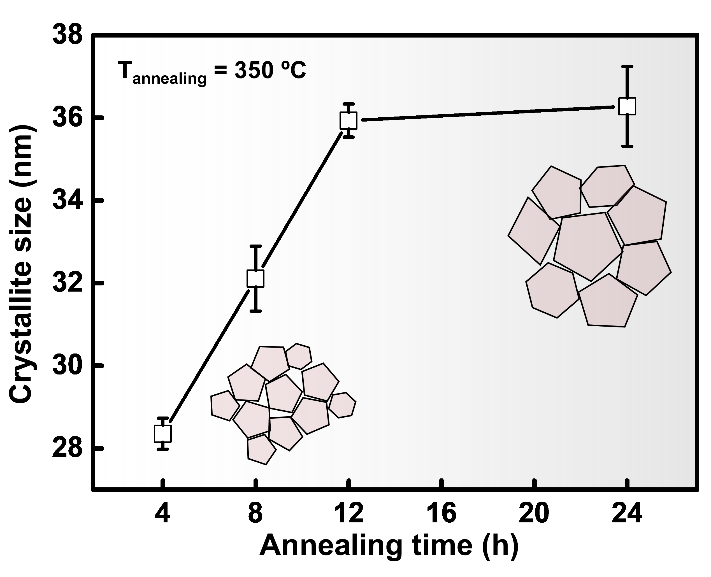


**Figure S1**. Variations in crystallite size of the Mg_2_Si thin films as a function of annealing time at 350 ℃.


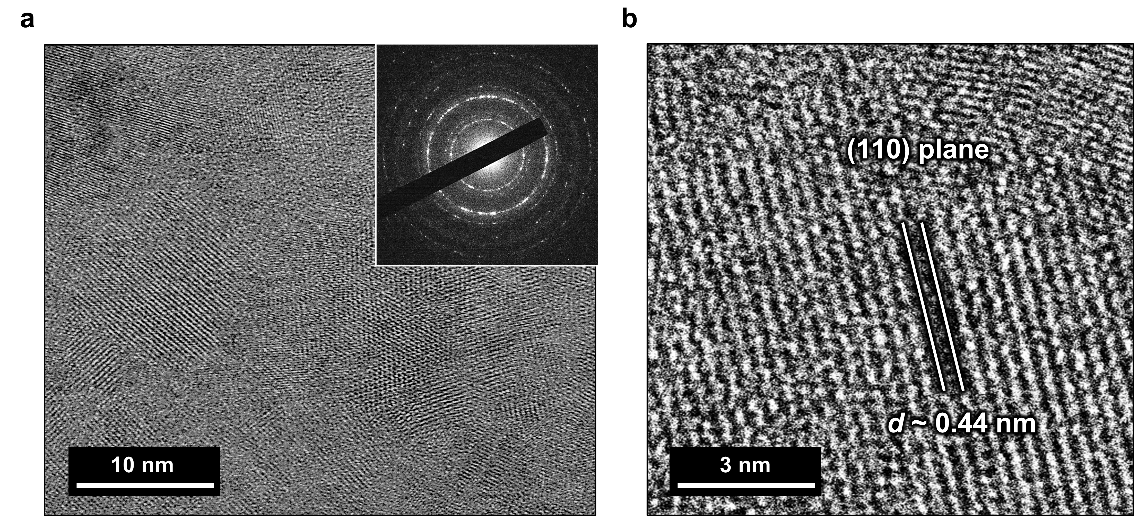


**Figure S2**. High-resolution transmission electron microscopy images of the Mg_2_Si thin films. a) Poly-nanocrystalline phase of the Mg_2_Si thin films annealed at 350 ℃ for 8 h (Inset: selected area electron diffraction patterns). b) Interplanar spacing of (110) planes of the Mg_2_Si thin films (350 ℃, 12 h).


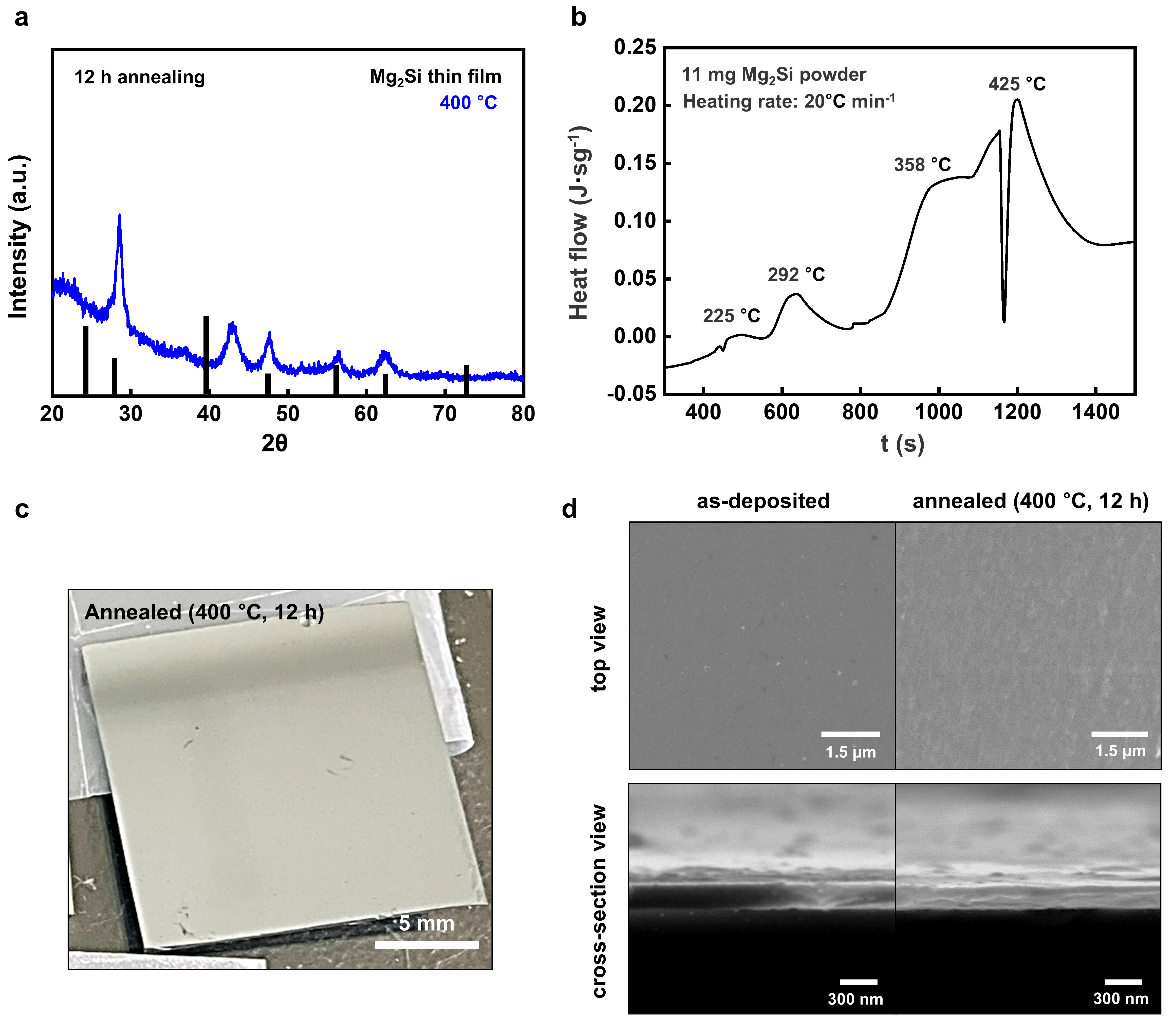


**Figure S3**. Annealing effect on crystal property and thin film morphology. a) X-ray diffraction patterns of the Mg_2_Si thin films annealed at 400 °C for 12 h. b) Differential scanning calorimetry curve of the Mg_2_Si thin films of heating rate of 20 °C min⁻^1^. c) Photograph of annealed Mg_2_Si thin film (400 °C, 12 h) deposited on Si wafer. d) SEM images of surface (top row) and cross-section images (bottom row) of the as-deposited (left) and annealed (400 °C, 12 h; right) Mg_2_Si thin films.


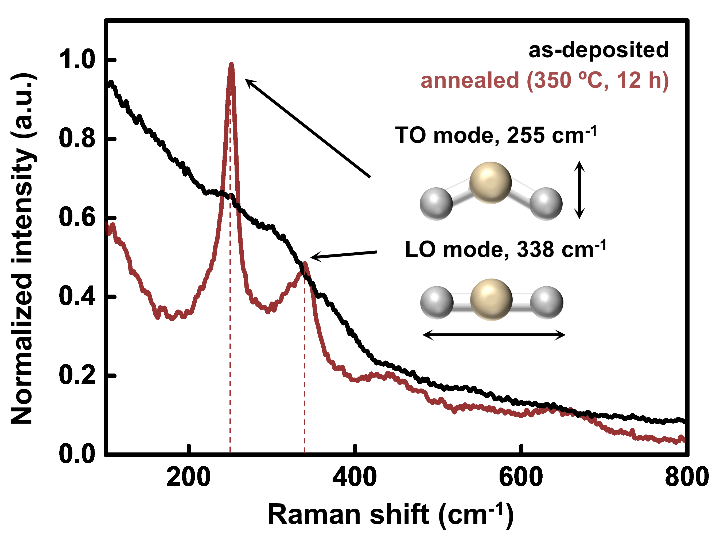


**Figure S4**. Raman spectrum of as-deposited Mg–Si (black) and annealed Mg_2_Si thin films (350 ℃, 12 h) (wine).


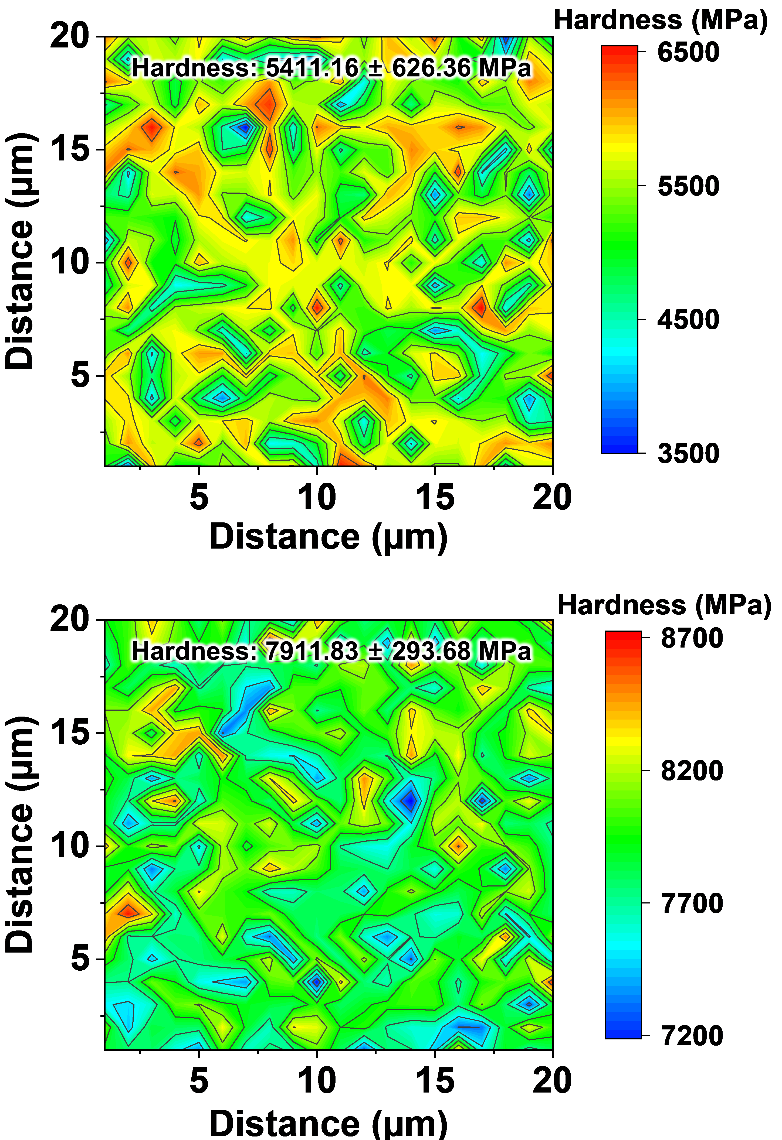


**Figure S5**. Hardness mapping of as-deposited Mg–Si and annealed Mg_2_Si thin films (350 ℃, 12 h) measured by using nanoindentation.


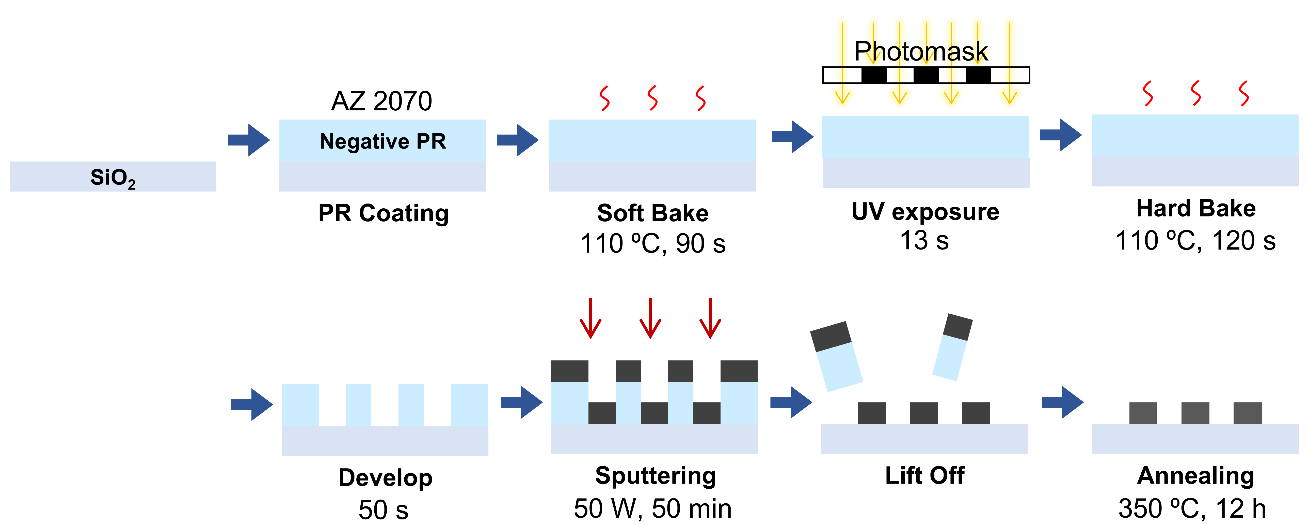


**Figure S6**. Fabrication process of the Mg_2_Si thin film pattern arrays for dissolution test.


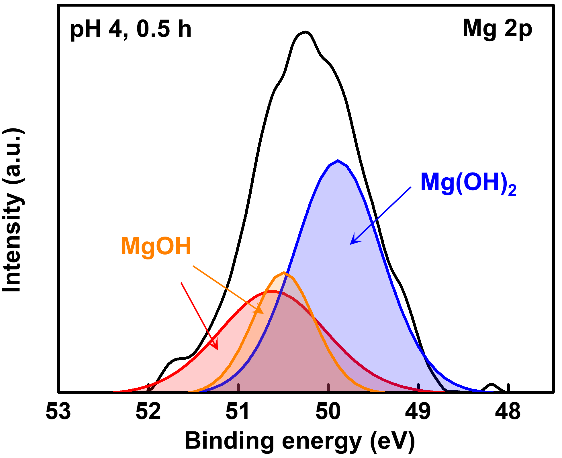


**Figure S7**. High-resolution deconvoluted spectrum of Mg 2p of the Mg_2_Si thin films immersed in pH buffer solution (pH 4) at 37 ℃ for 0.5 h.


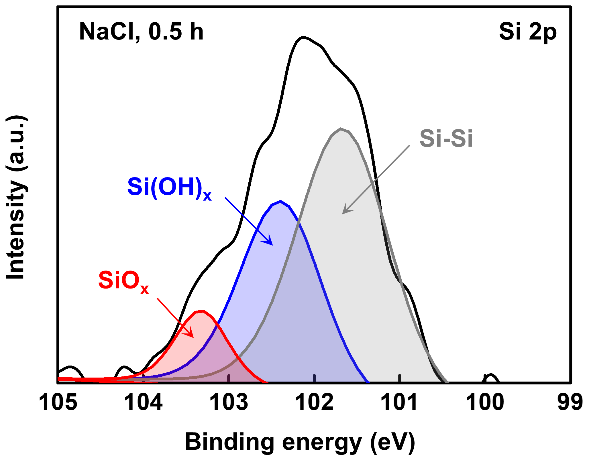


**Figure S8**. High-resolution deconvoluted spectrum of Si 2p of the Mg_2_Si thin films immersed in 1 M of NaCl solution at 37 ℃ for 0.5 h.


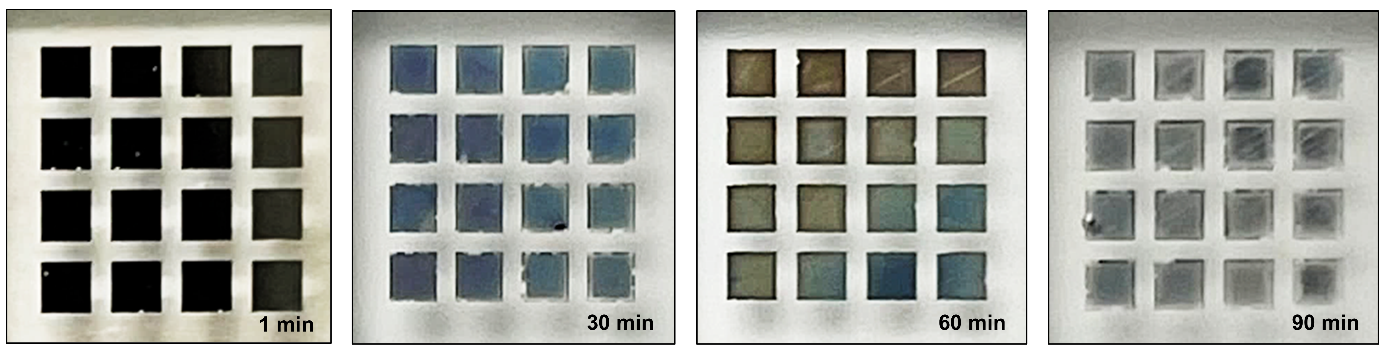


**Figure S9**. Photographic images showing biodegradation process of the Mg_2_Si thin film pattern arrays in phosphate-buffered saline (PBS; 37 ℃, pH 7.4).


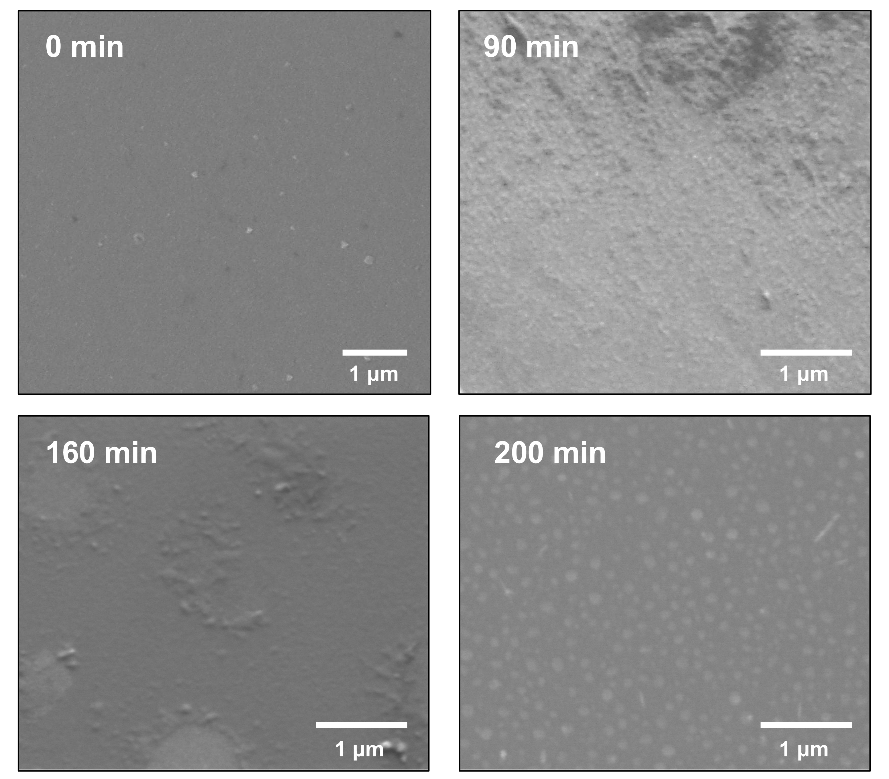


**Figure S10**. SEM images of the Mg_2_Si thin films under PBS (pH 7.4, 37 °C) immersion for 0, 90, 160, 200 min.


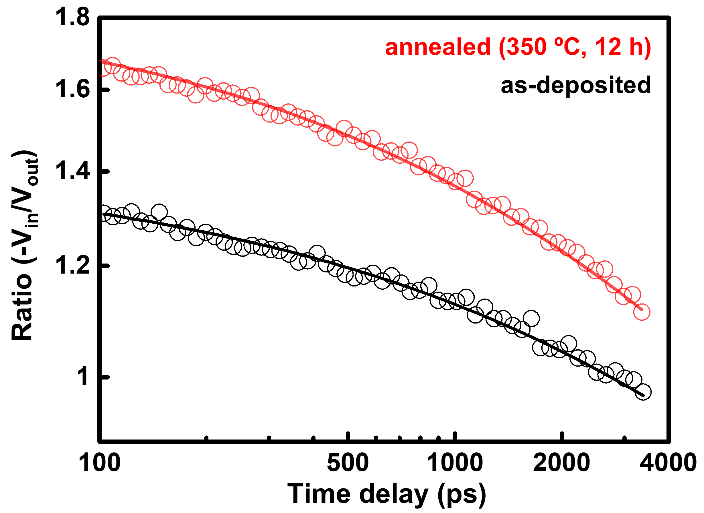


**Figure S11**. Experimental (dots) and fitted (lines) results of time domain thermoreflectance (TDTR) measurement to verify the thermal conductivity of the Mg_2_Si thin films for as-deposited (black) and annealed (350 ℃, 12 h) (red) samples.


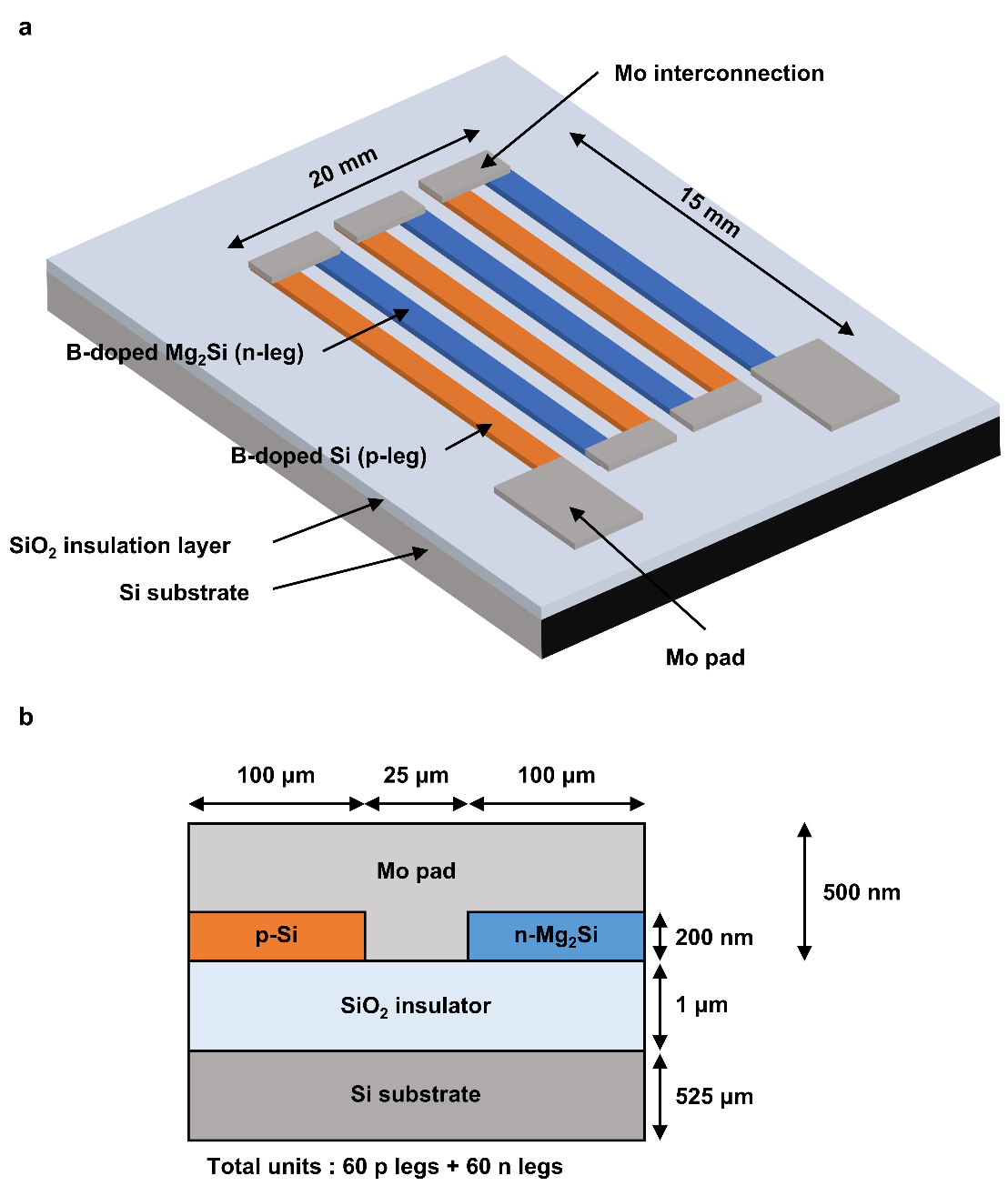


**Figure S12**. Exploded view and dimensions of B-doped Mg_2_Si thin film based thermoelectric generator (TEG). a) Exploded view and length dimensions of B-doped Mg_2_Si thin film-based TEG. b) Thickness and width dimensions of B-doped Mg_2_Si thin film-based TEG.


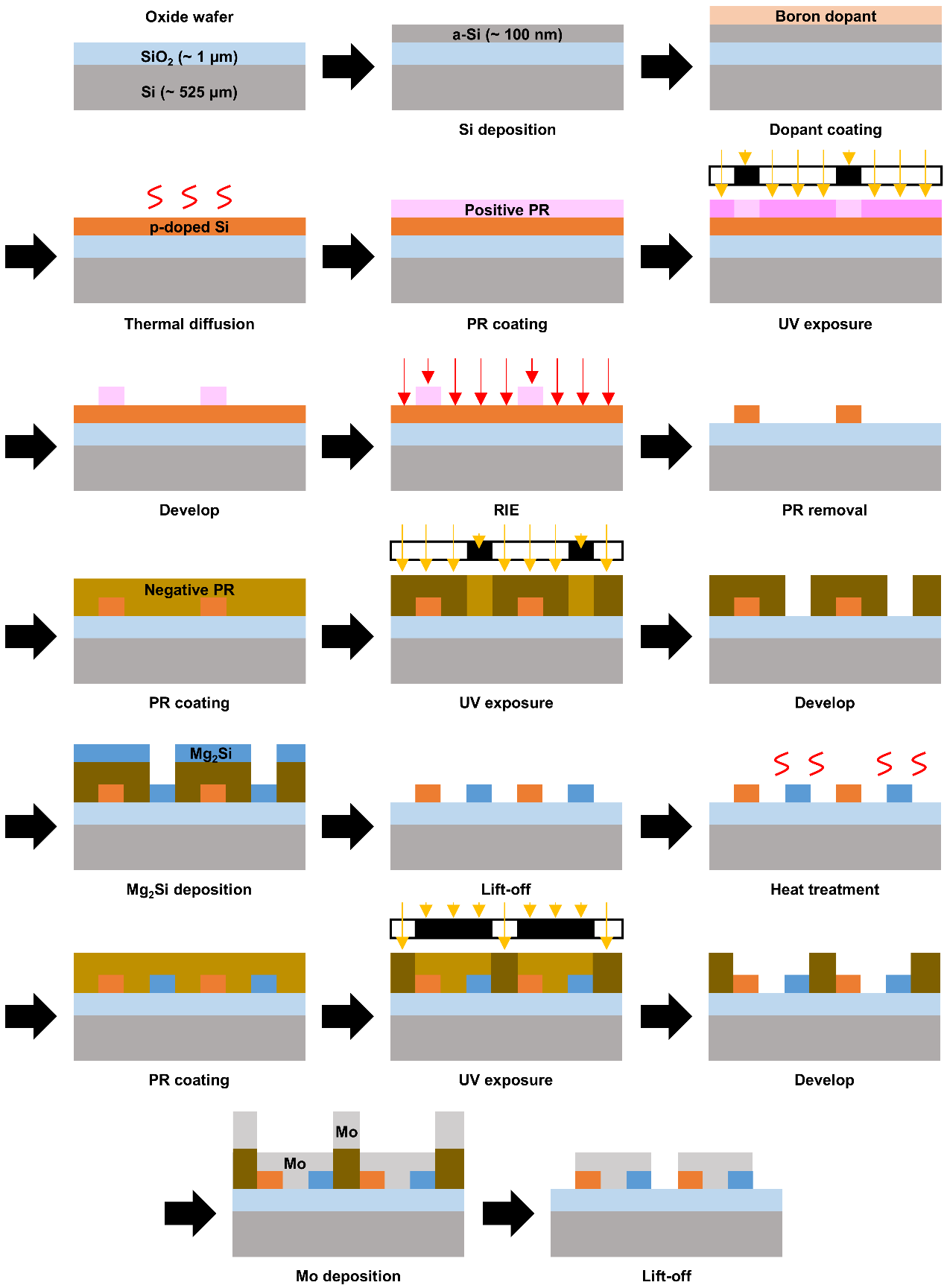


**Figure S13**. Fabrication procedure of B-doped Mg_2_Si thin film-based TEG.


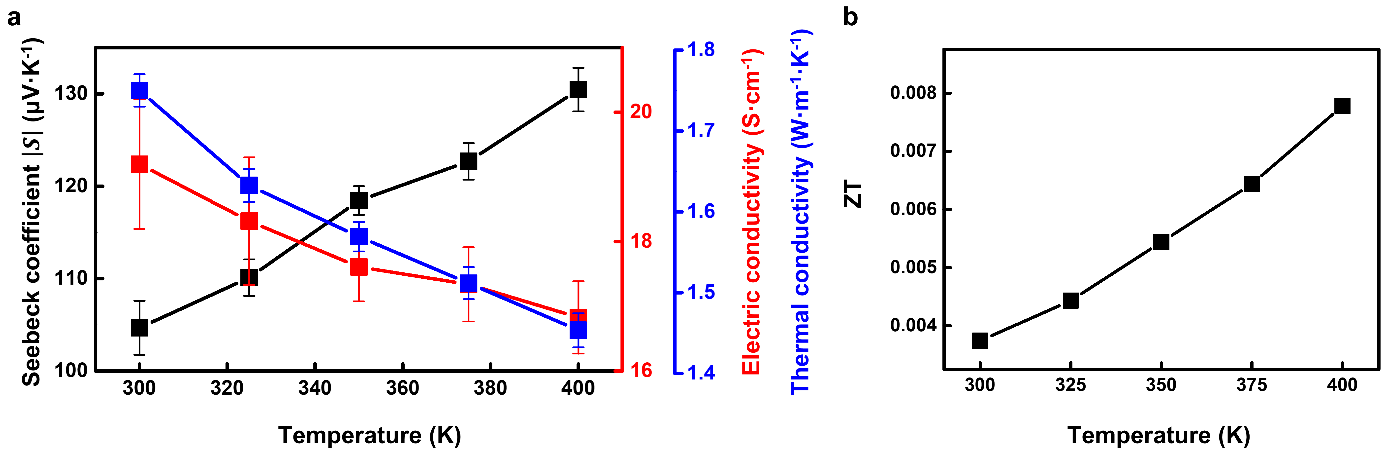


**Figure S14**. Thermoelectric characteristics of non-doped Mg_2_Si. a) Temperature-dependent Seebeck coefficient (black), electrical conductivity (red), and thermal conductivity (blue) of non-doped Mg_2_Si thin film. b) Calculated figure of merit (ZT) values of the non-doped Mg_2_Si thin film as a function of temperature.


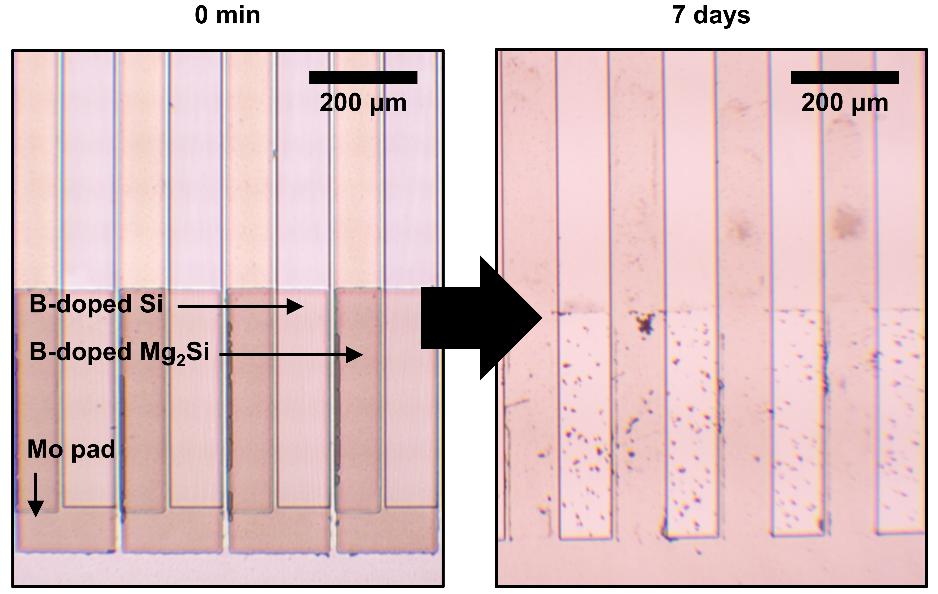


**Figure S15**. Additional sequential OM images during degradation of the B-doped Mg_2_Si thin film-based TEG in PBS (37 ℃, pH 7.4).


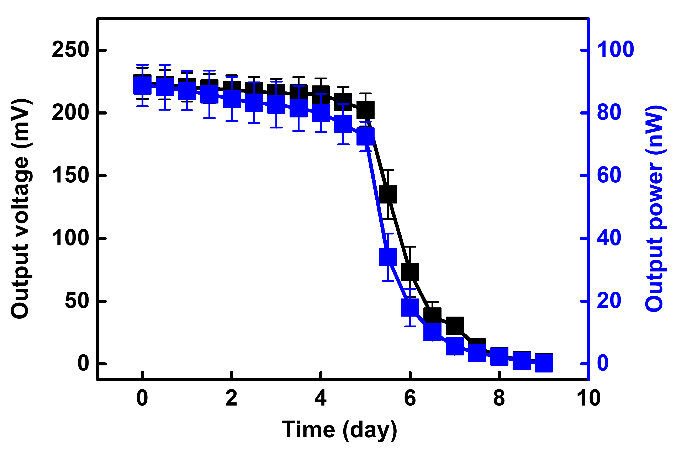


**Figure S16.** Output degradation of the B:Mg_2_Si thin film-based TEG with PBAT encapsulation.


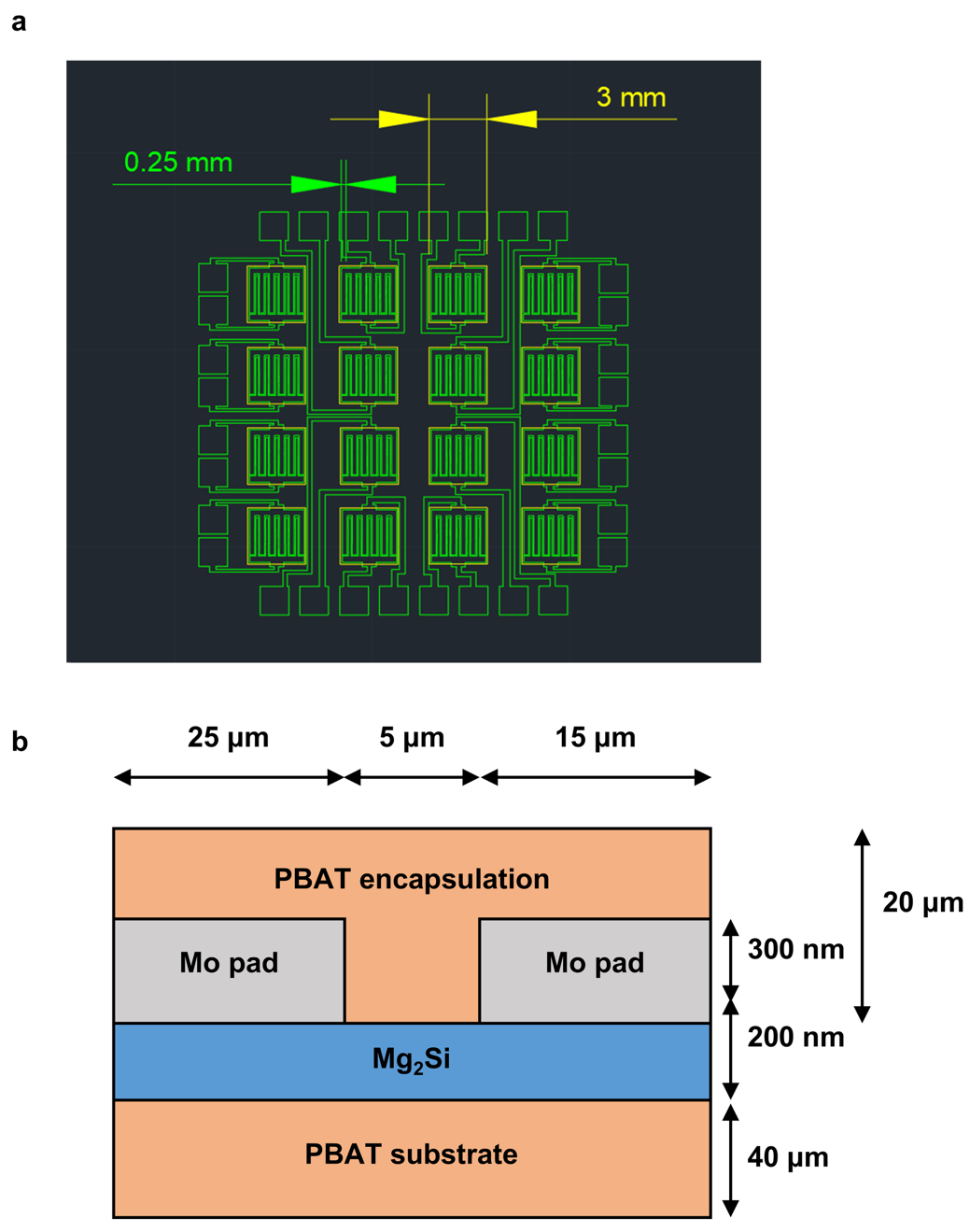


**Figure S17**. Exploded view and dimensions of the Mg_2_Si thin film photosensor. a) Exploded view and length dimensions of the Mg_2_Si thin film photosensor. b) Thickness and width dimensions of the Mg_2_Si thin film photosensor.


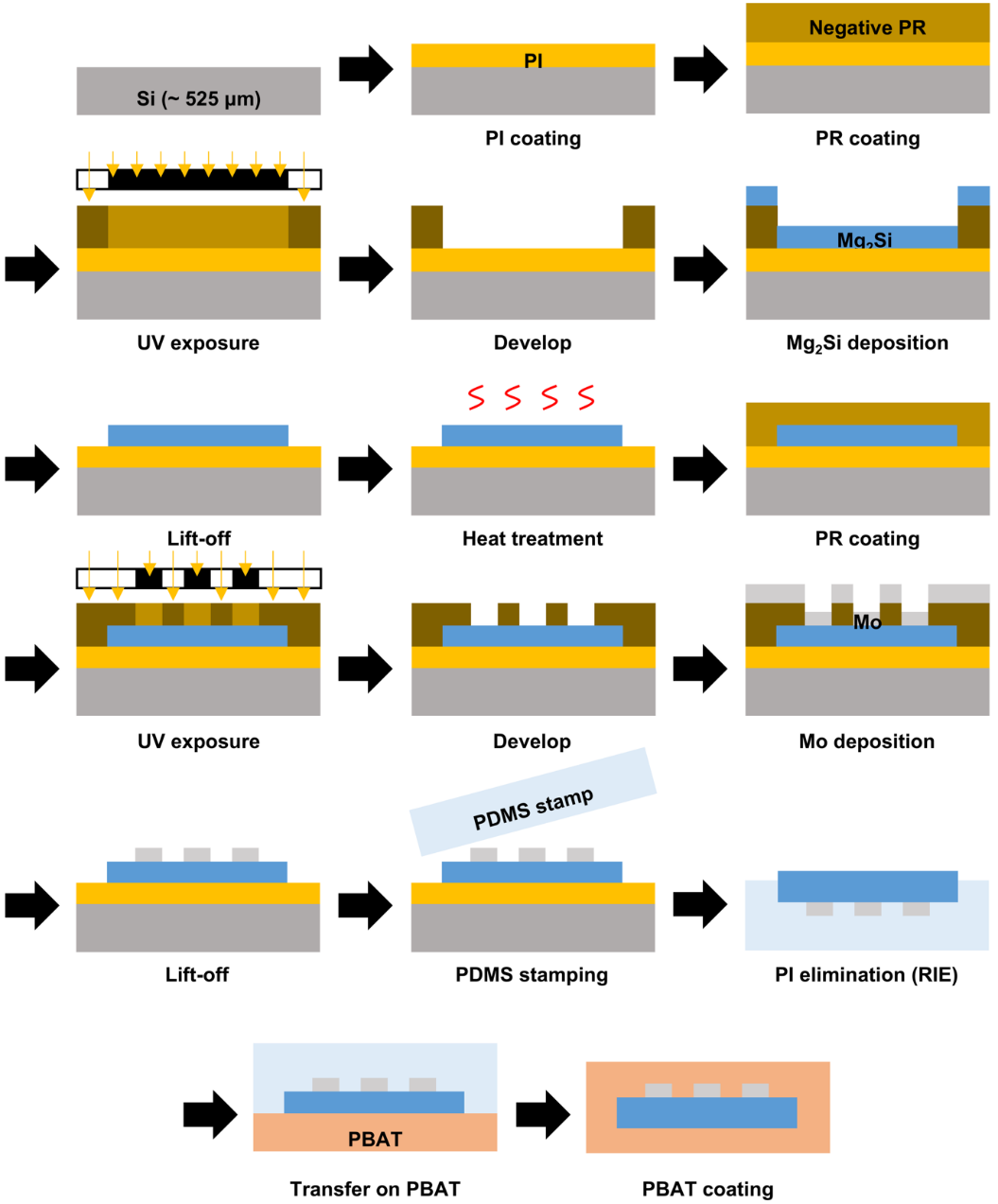


**Figure S18**. Fabrication procedure of Mg_2_Si thin film photosensor.


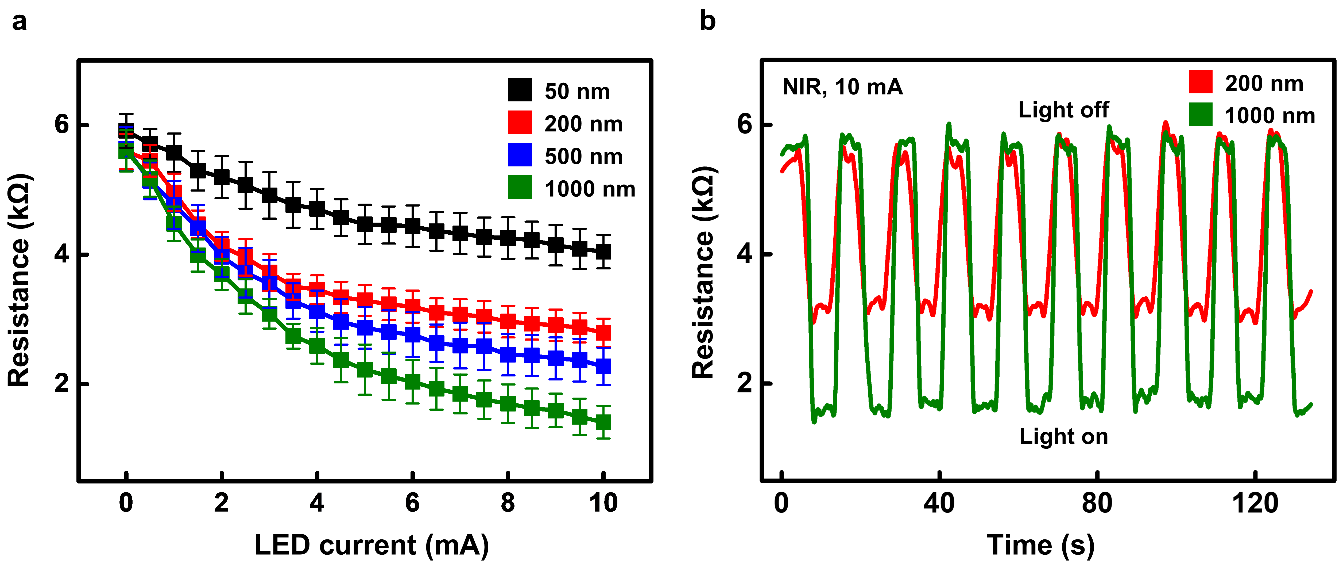


**Figure S19**. NIR (940 nm) sensitivity of the Mg_2_Si thin film-based optoelectronic device with different thickness. a) LED current–dependent resistance changes of 50 nm (black), 200 nm (green), 500 nm (red), 1000 nm (blue) thick Mg_2_Si under NIR. b) On/off switching of the device with 200 nm (black) and 1000 nm (red) thick Mg_2_Si under NIR at 10 mA of LED current.


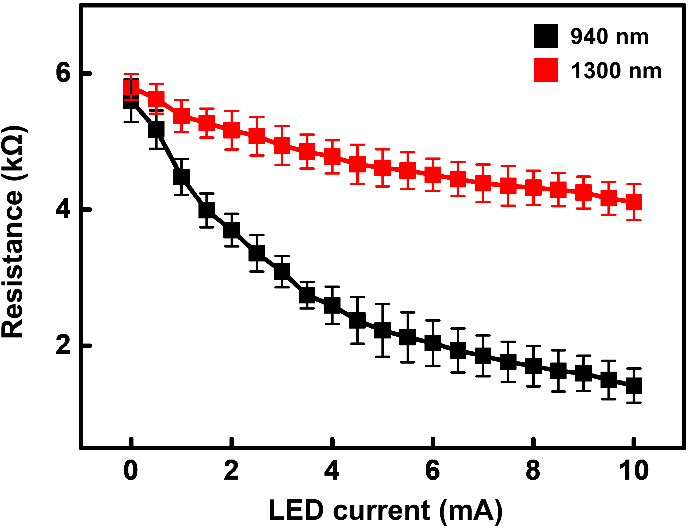


**Figure S20**. Long wavelength sensitivity of the Mg_2_Si thin film-based optoelectronic device. LED current–dependent resistance changes of 1000 nm thick Mg_2_Si under 940 nm (black) and 1300 nm (red) light illumination.


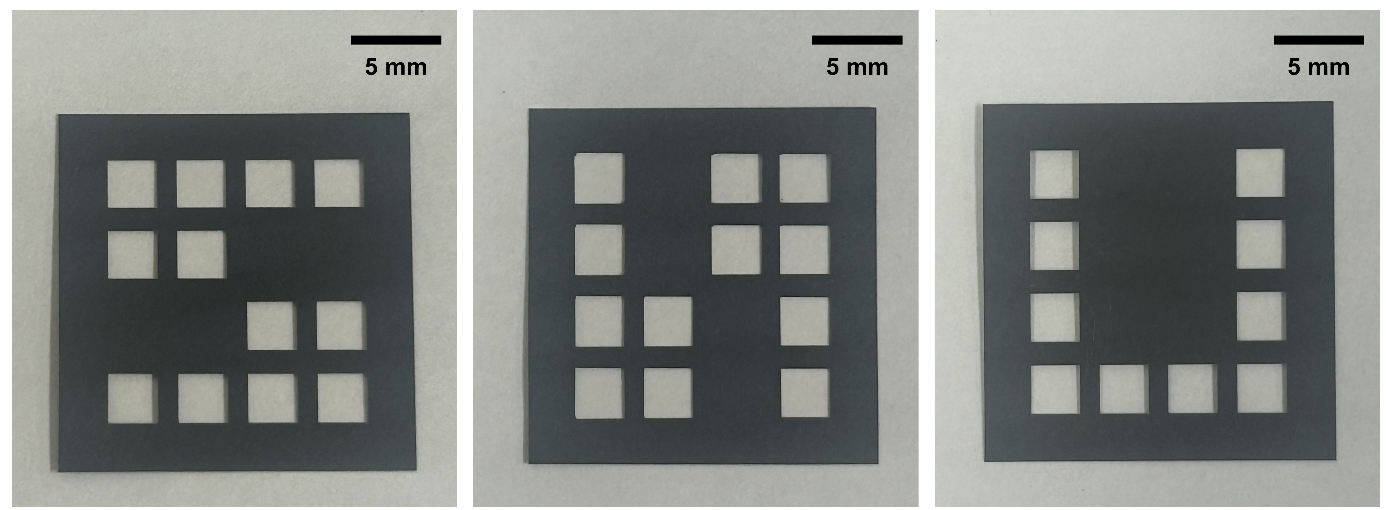


**Figure S21**. Shadow masks for photo-imaging of the Mg_2_Si thin film photosensor.


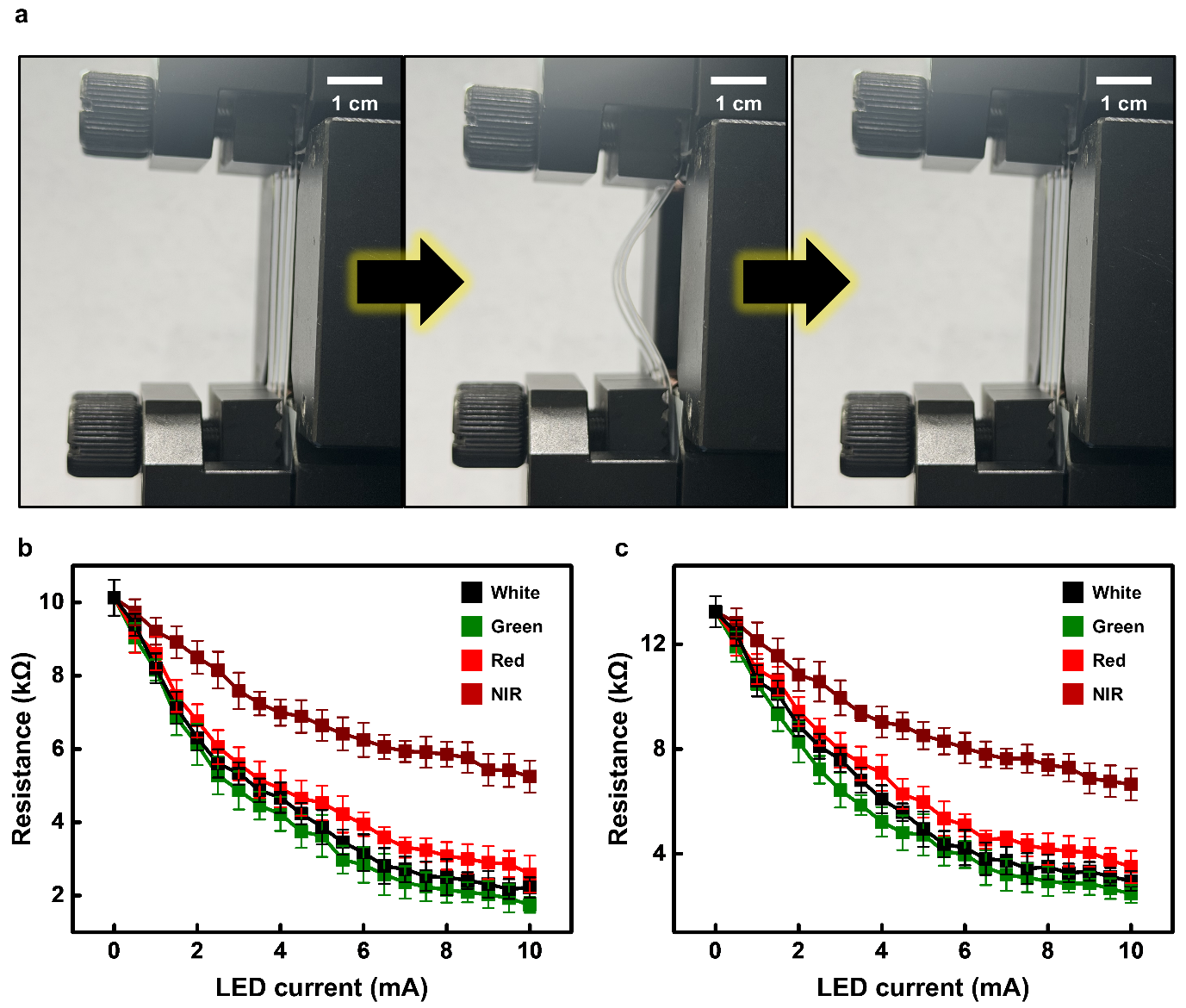


**Figure S22**. Bending durability of the Mg_2_Si thin films. a) Photograph of bending test of the Mg_2_Si thin film-based optoelectronic devices (4 cm × 0.25 cm × 200 nm). LED current–dependent resistance changes under white (400 to 800 nm, black), green (527 nm, green), red (630 nm, red), and NIR (940 nm, wine) b) before and c) after bending cycles of 10000 with a bending radius of 1 cm.


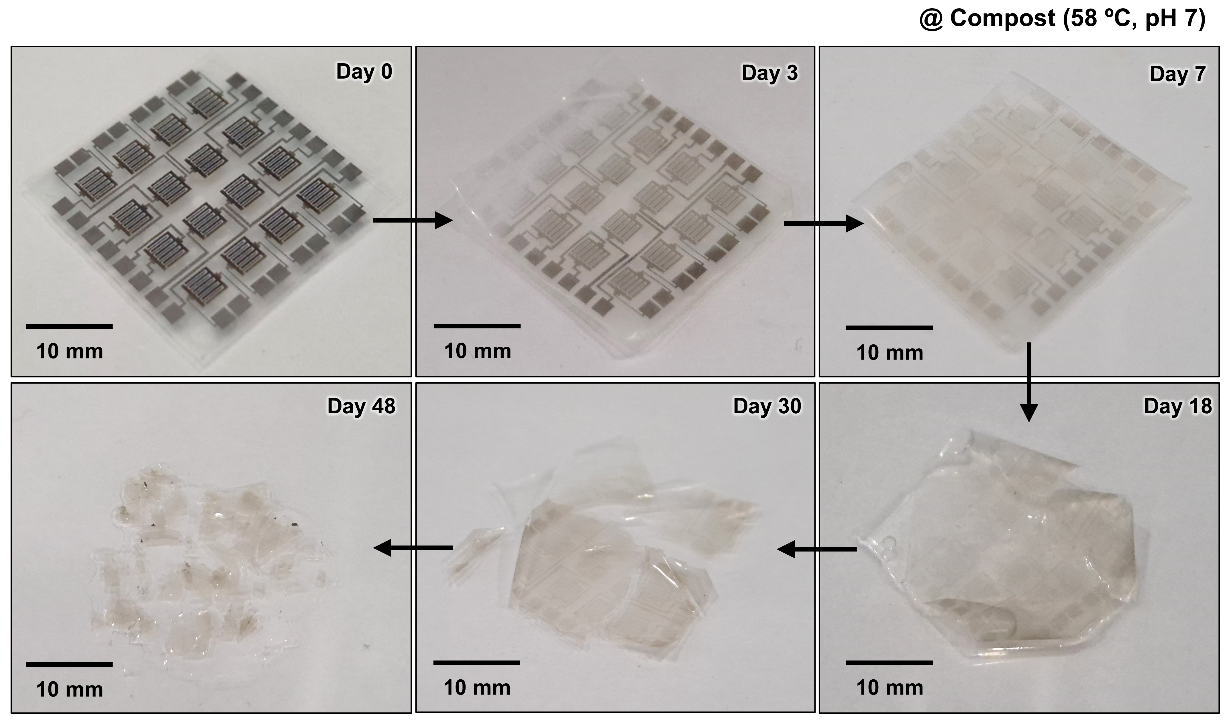


**Figure S23**. Photographic image sequence showing hydrolysis reaction of the Mg_2_Si thin film photosensor in composting environment (58 ℃, pH 7) under immersion time.

| \| Ref. \| Method  Dopant (concentration) \| Forms of specimen \| Electrical conductivity  (S cm⁻^1^) \| Total thermal conductivity  (W m⁻^1^K⁻^1^) \| Figure of merit, ZT (temperature range) \| \| --- \| --- \| --- \| --- \| --- \| --- \| \| [1] \| Spark plasma sintering  Sb doping (0.5 at%) \| Bulk cylinders \| 1 x 10^3^ \| 5.8 \| 0.15 (400 K) \| \| [2] \| Spark plasma sintering  B doping (0.02 M) \| Bulk plates \| 1 x 10^1^ \| 2.8 \| 0.015 (400 K) \| \| [3] \| Spark plasma sintering  B doping (0.75 at%) \| Bulk cylinders \| 3.6 x 10^2^ \| 3.9 \| 0.21 (400 K) \| \| [4] \| Plasma activated sintering (PAS)  (undoped) \| Bulk plates \| 2 x 10^2^ \| 4.1 \| 0.25 (400 K) \| \| [5] \| Hot press  Sn doping (Mg:Si:Sn=2:1-x:x) \| Bulk plates \| 1.3 x 10^3^ \| 2.31 \| 0.62 (400 K) \| \| This study \| Sputtering and annealing  B doping (1 at%) \| Thin films on SiO_2_, Si, PBAT \| 7.6 x 10^1^ \| 1.45 \| 0.034 (400 K) \| |
| --- | --- | --- | --- | --- | --- | --- | --- | --- | --- | --- | --- | --- | --- | --- | --- | --- | --- | --- | --- | --- | --- | --- | --- | --- | --- | --- | --- | --- | --- | --- | --- | --- | --- | --- | --- | --- | --- | --- | --- | --- | --- | --- |

**Table S1**. Comparison of materials and devices characteristics from previous studies on Mg_2_Si-based thermoelectric device.
